# A Messaging App Empowering Lifestyle Modification in Chronic Kidney Disease (LINE Official Account “Kidney Lifestyle”): Platform Development and Usability Study

**DOI:** 10.2196/73935

**Published:** 2025-11-26

**Authors:** Chun-Yi Ho, Deborah Siregar, Miaofen Yen, Junne-Ming Sung, Ming-Cheng Wang, Wei-Hung Lin

**Affiliations:** 1 Department of Nursing, College of Medicine, National Cheng Kung University, No. 1, Daxue Rd, East Dist, Tainan City, 701401, Taiwan, 886 6-235-3535 ext 5823; 2 Division of Nephrology, Department of Internal Medicine, National Cheng Kung University Hospital, College of Medicine, National Cheng Kung University, Tainan City, Taiwan; 3 Division of General Internal Medicine, Department of Internal Medicine, National Cheng Kung University Hospital, College of Medicine, National Cheng Kung University, Tainan City, Taiwan

**Keywords:** chronic kidney disease, dyadic empowerment, lifestyle modification, digital health, instant messaging, usability testing, LINE app

## Abstract

**Background:**

Regular lifestyle modification is crucial for chronic kidney disease (CKD) management; yet, older patients often struggle to sustain behavior change and rely on support from their significant others such as family caregivers or partners. In such cases, both members of the dyad require accessible, jointly usable tools to maintain healthier behaviors over time. Given the ubiquity of instant messaging platforms, a digital intervention delivered via such a platform offers strong potential to empower CKD dyads in active lifestyle modification.

**Objective:**

Guided by the Digital Dyadic Empowerment Framework, this study aimed to develop, optimize, and test the usability of a digital platform named “Kidney Lifestyle,” using the LINE Official Account (OA) and an integrated extended app to facilitate collaborative lifestyle modification among CKD dyads.

**Methods:**

We adopted a three-phase Agile-based development cycle: (1) iterative development and trial use, (2) heuristic evaluation, and (3) usability testing. In phase 1, the platform prototype was codeveloped with health care professionals and trialed by CKD dyads who provided feedback on interface clarity, ease of use, acceptance, intention to continue usage, and overall satisfaction. In phase 2, multidisciplinary experts conducted heuristic evaluations, rating compliance with Nielsen’s 10 usability principles and suggesting improvements. In phase 3, experienced CKD dyads from phase 1 performed 6 representative tasks using the platform. Task success rates, completion times, and operational errors were recorded, and usability perceptions were assessed using the After-Scenario Questionnaire (1‐7) and the System Usability Scale (0‐100).

**Results:**

In phase 1, 10 CKD dyads (19 individuals) reported high acceptance (mean overall satisfaction 4.1/5), valuing real-time interaction, convenient health data monitoring, and educational resources. In phase 2, 5 experts found high usability compliance (89%‐93%) but noted navigation complexity and the need for more interactive feedback. In phase 3, usability testing with 5 dyads showed high task success (60%‐100%) and short completion times (1‐5 minutes). Extended app tasks used for structured self-monitoring achieved higher satisfaction, reflecting simpler navigation than tasks within the LINE OA (mean After-Scenario Questionnaire 5.64 vs 3.87). Navigation difficulties within LINE OA were likely due to multilayered menus and limited customization. The average System Usability Scale was 67.5, indicating marginally acceptable usability.

**Conclusions:**

The LINE-based digital dyadic empowerment platform “Kidney Lifestyle*”* demonstrated promising usability and engagement. It has clinical potential to improve CKD control by extending health education, enabling continuous self-monitoring, and allowing clinicians to track patients’ daily living conditions. To enhance effectiveness, future work should include a larger-scale feasibility trial while pursuing ongoing platform optimization, specifically by simplifying navigation pathways, adding a return option, and improving interactive feedback. The platform is now publicly accessible via LINE ID search, as provided in phase 1 results.

## Introduction

### Background

Chronic kidney disease (CKD) poses a serious burden to global health, affecting approximately 9.1% of the population or around 700 million people worldwide [1]. In Taiwan, the increasing prevalence of end-stage kidney disease has escalated health care costs, imposing substantial strain on the National Health Insurance system [2]. Effective CKD management can delay disease progression to end-stage kidney disease, reduce the risk of complications, and enhance patients’ quality of life [3,4]. However, significant barriers remain, including insufficient continuous monitoring, limited patient education, and restricted access to health care providers [5,6].

CKD primarily affects older adults, especially those older than 60 years with lower educational levels, underscoring the need for tailored interventions for this group [7-10]. Digital health interventions (DHIs) offer unique opportunities to maintain patient engagement and provide tailored health education, particularly for older adults to improve autonomy and overall well-being [11]. With the widespread use of digital and mobile technologies, DHIs have become a key part of chronic care, supporting various health needs effectively [12]. Previous studies have shown that DHIs can improve self-management among patients with CKD [13]. This potential has led to the ongoing development of diverse tools—ranging from smartphone apps to web-based platforms—designed to support CKD and dialysis self-care [14-17].

Building on these advances, instant messaging platforms have emerged as promising tools for ubiquitous CKD management support. LINE, in particular, has gained significant popularity in Asia due to its user-friendly interface and broad demographic reach, effectively engaging users of all ages [18]. In Taiwan, LINE dominates the instant messaging market with more than 90% market share, making it a highly suitable platform for health communication [19]. The LINE app has already demonstrated potential in facilitating health education and self-management among middle-aged patients with CKD [20,21]. However, a LINE-based platform promoting lifestyle modification for older (older than 60 years) patients with CKD and their caregivers remains scarce. The extensive user base of LINE presents a unique opportunity to deliver cost-effective DHIs supporting multifaceted CKD management for this older population through a LINE Official Account (LINE OA).

### Previous Work

In our previous work, we developed the Digital Dyadic Empowerment Framework (DDEF) as a conceptual and practical guide for designing DHIs that empower both patients with CKD and their significant others (SOs) (eg, spouses and children) to sustain lifestyle modification. The framework identifies 5 core domains of dyadic empowerment, including *perceived control*, *understanding*, *adaptation*, *dyadic efficacy*, and *dyadic support*. It further proposes 5 digital strategies: *digital education*, *digital communication*, *digital monitoring*, *digital feedback*, and *digital analysis*, through which DHIs can be designed to strengthen these dyadic empowerment domains. Together, these domains and strategies specify *what* to empower in the dyad and *how* to operationalize dyadic empowerment through concrete digital features, providing a structured pathway for designing and evaluating DHIs that support collaborative CKD self-management.

### Objectives

This study aimed to develop, evaluate, and optimize the usability of the LINE-based digital platform “Kidney Lifestyle” for CKD dyads. The platform was directly developed under the guidance of the DDEF, applying its 5 digital strategies to strengthen dyadic empowerment and promote sustainable lifestyle modification among CKD dyads. This research specifically investigates the platform’s usability, user acceptance, and potential clinical application in CKD management.

## Methods

### Study Design

**Figure 1.**
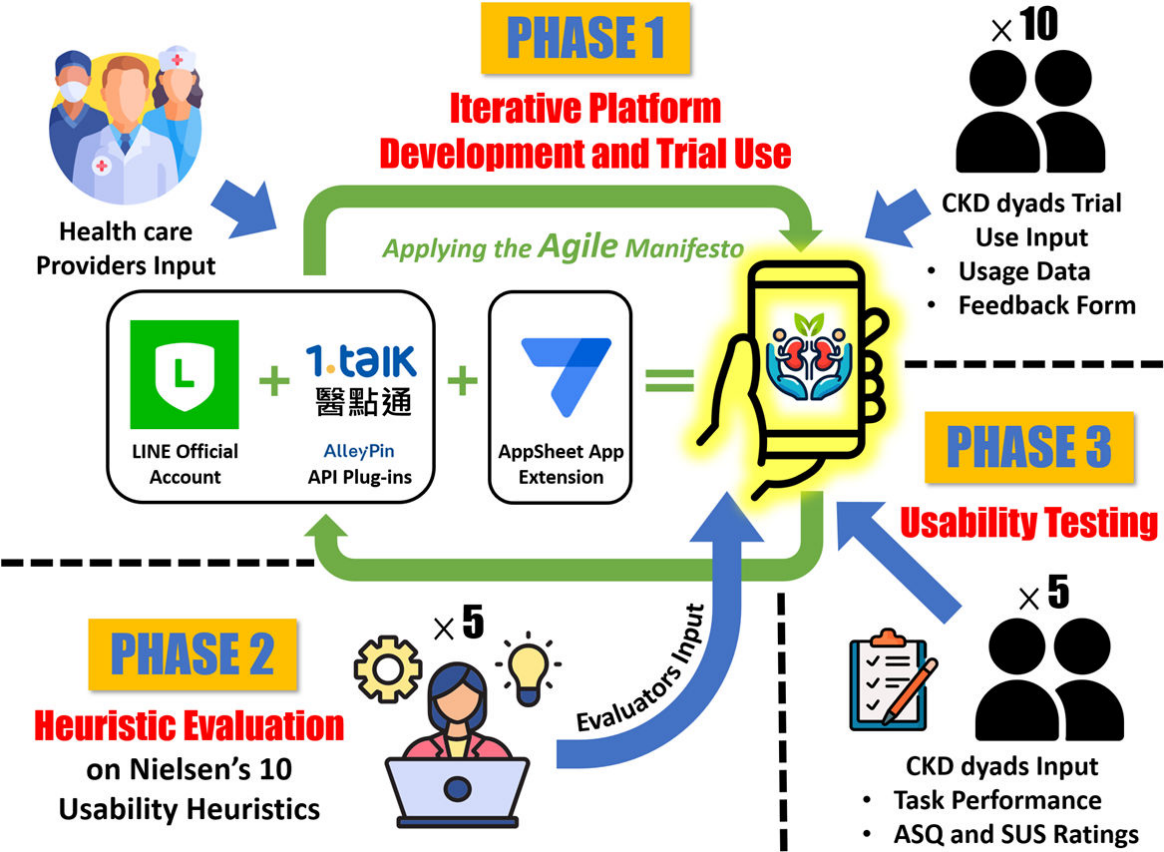
A visualization of the 3-phase iterative system development cycle. Blue arrows denote the input provided by the participants from each phase. ASQ: After-Scenario Questionnaire; CKD: chronic kidney disease; SUS: System Usability Scale.

Following the guidelines outlined by Kushniruk and Patel [22], a 3-phase iterative system development cycle was used to develop and test our LINE-based digital dyadic empowerment platform. These phases included (1) iterative development and trial use, (2) heuristic evaluation, and (3) usability testing (Figure 1). An Agile project management methodology was applied throughout the development cycle to manage initial uncertainties and facilitate continuous stakeholder interaction [23]. During each phase, participants identified usability issues, which guided subsequent refinements of the digital platform. The details of each phase are described in the following sections.

### Phase 1: Iterative Development and Trial Use

Phase 1 focused on developing the platform, especially the functionalities and content. Summarizing retrospectively, the platform development process involved the following four iterative steps: (1) platform establishment, (2) technical partner collaboration, (3) extended platform development, and (4) all platform integration and intervention design.

#### Step 1: Platform Establishment

A regular LINE OA was first set up to explore its basic functionalities, applying two functions aligned with 3 DDEF digital strategies: (1) chat: “Digital Feedback” and “Digital Communication,” and (2) multimedia and multipage messages: “Digital Education.” For example, CKD-related information (eg, symptoms, staging, and risk factors) could be delivered through autoresponse messages triggered by specific keywords. However, such keyword-based use was inconvenient and insufficient for promoting lifestyle modification among CKD dyads, as prior medical applications of LINE OAs mainly focused on clinic operations (eg, appointment scheduling and reminders) rather than ongoing lifestyle support [24]. These limitations highlighted the need for integration with third-party application programming interface (API) plug-ins to enable more advanced functionalities.

#### Step 2: Technical Partner Collaboration

AlleyPin Interactive Ltd Co is a LINE-certified Gold-level technical partner in the health care industry, providing API technical resources to meet diverse needs. Their cloud-based doctor-patient interaction management system, “DoctorConnect 1.Talk,” when integrated with the team’s LINE OA, can provide more advanced and user-friendly functionalities, including (1) service menu setup: it allows up to 8 buttons with multilayered structures for resource integration, beyond the 6-button limit of LINE OA; (2) enhanced multimedia and multipage messages: multiple buttons can be set within a single message, enabling users to access tailored health information via menu clicks without manual text input; and (3) case tracking system: it supports member management, scheduled “Push” messages, and interaction through AlleyPin’s independent backend.

The service menu content and layout were designed not only by following the DDEF but also in consultation with a health education nurse from a university-affiliated hospital and 2 nephrologists, ensuring the platform met clinical needs. Through the integration of LINE OA and AlleyPin Interactive’s API plug-in, our platform now effectively adopted 3 digital strategies—“Digital Education,” “Digital Feedback,” and “Digital Communication*”*—to support CKD dyads. To further apply the other 2 digital strategies—“Digital Monitoring” and “Digital Analysis,” an extended app was developed and integrated with the LINE OA in the next step.

#### Step 3: Extended App (AppSheet) Development

After exploring various options and evaluating alternative platforms, the team discovered that Google’s AppSheet platform could effectively compensate for the current platform’s shortcomings and integrate with LINE OA. AppSheet is a platform for developing mobile and web apps that do not require programming code and can integrate with cloud spreadsheets or databases such as Google Drive and Office 365 as data sources [25]. The team immediately began developing the app prototype and conducted iterative testing.

#### Step 4: Platform Integration and Intervention Design

As the digital platform was not selected during the development of DDEF, the team, after the platform selection and development process outlined in steps 1-3, reviewed the correspondence between the DDEF and the functions provided by this integrated digital platform. Based on this, interventions were designed to promote lifestyle modification for CKD dyads.

#### Platform Trial Use

After establishing our digital platform prototype, the team invited 10 early-stage CKD (stages 1-3) dyads receiving regular treatment at a university hospital to use the platform in their daily lives. Besides assisting dyads in installing the digital platform on their smartphones, a user manual (Multimedia Appendix 1) was provided for each dyad to learn the operations at home. At the time of enrollment, operational instructions for various functions were provided by a research assistant (CYH) according to the manual.

A user feedback form (Multimedia Appendix 2) was provided to be filled out by the dyads at least 1 month apart during their next clinic visit. For each of the 6 major functions, the form assessed the following five elements: (1) interface clarity, (2) ease of operation, (3) acceptability: like or not, (4) intention to continue usage, and (5) satisfaction rating from 1 to 5 (Table 1). A brief interview was conducted with each dyad to obtain further feedback. The platform was iteratively optimized based on both system usage data and user feedback.

**Table 1.** Content of user feedback form for phase 1 dyadic trial use^a^.

Element (item number) and question			Type
Interface clarity (N-1)			
		Are there any unclear elements on the screen?	Yes/No
		If yes, please specify.	Short answer
Ease of operation (N-2)			
		Is this function easy to use?	Yes/No
		If not, please explain why.	Short answer
Acceptability (N-3)			
		Do you like this function?	Yes/No
		Tell us what you like about this function?	Short answer
		If you dislike this function, how can we improve?	Short answer
Intention to continue usage (N-4)			
		Will you keep using this function?	Yes/No
		Why or why not?	Short answer
Satisfaction (N-5)			
		Please rate your satisfaction with this function on a scale from 1 to 5.	5-Point Scale
Previous experience (7-1)			
		Have you ever used any other apps or websites for managing chronic kidney disease?	Yes/No
		If yes, what did you like or dislike about them?	Short answer
General platform satisfaction (7-2)			
		What is your overall satisfaction rating with our platform?	5-Point Scale
		Will you continue to use our platform for chronic kidney disease management?	Yes/No

a Items N-1 to N-5 are inquired repeatedly for each of the 6 functions of the platform, resulting in a total of 30 questions. Items 7‐1 and 7‐2 are general questions asked once, adding 2 more questions. Therefore, the total number of questions in this form is 32.

### Phase 2: Heuristic Evaluation

To further examine whether our digital platform is user-friendly in its design, a heuristic evaluation involving 5 evaluators with backgrounds in health care or information engineering was conducted. Evaluators tried out the digital platform and assessed how well each of the 6 major functions in the interface design complies with Nielsen’s 10 usability principles (10 usability heuristics) [26].

The team referred to Moran and Gordon’s [27] suggested heuristic evaluation steps and designed an evaluation form based on their published *Heuristic Evaluation Handbook*. Considering that the digital platform’s access is not limited by time or space and acknowledging the need for evaluators to independently conduct the evaluation, the team opted for evaluators to fill out Google Forms for remote participation.

The form begins by inviting evaluators to join our digital platform on LINE and set up the initial settings. Evaluators were then asked to use the platform for 3 days. Specifically, they explored the buttons’ functionalities and information on the LINE OA service menu and the extended app, similar to the real-use scenarios in phase 1 dyadic trial use. Before starting the evaluation, evaluators are invited to visit relevant websites to familiarize themselves with the content of Nielsen’s 10 usability heuristics [28]. The explanations and the assessment questions for each principle are shown in Table 2.

**Table 2.** Explanation of 10 usability heuristics and assessment questions^a^.

Heuristic	Explanation	Assessment questions
Principle 1: Visibility of System Status	The design should always keep users informed about what is going on through appropriate feedback within a reasonable amount of time.	Does the design clearly communicate its state? Is feedback presented quickly after user actions?
Principle 2: Match Between System and the Real World	The design should speak the users’ language. Use words, phrases, and concepts familiar to the user, rather than internal jargon. Follow real-world conventions, making information appear in a natural and logical order.	Will the user be familiar with the terminology used in the design? Do the design’s controls follow real-world conventions?
Principle 3: User Control and Freedom	Users often perform actions by mistake. They need a clearly marked “emergency exit” to leave the unwanted action without having to go through an extended process.	Does the design allow users to go back a step in the process? Are exit links easily discoverable? Can users easily cancel an action? Is *Undo* and *Redo* supported?
Principle 4: Consistency and Standards	Users should not have to wonder whether different words, situations, or actions mean the same thing. Follow platform and industry conventions.	Does the design follow industry conventions? Are visual treatments used consistently throughout the design?
Principle 5: Error Prevention	Good error messages are important, but the best designs carefully prevent problems from occurring in the first place. Either eliminate error-prone conditions or check for them and present users with a confirmation option before they commit to the action.	Does the design prevent slips by using helpful constraints? Does the design warn users before they perform risky actions?
Principle 6: Recognition Rather Than Recall	Minimize the user’s memory load by making elements, actions, and options visible. The user should not have to remember information from one part of the interface to another. Information required to use the design (eg, field labels^b^ or menu items) should be visible or easily retrievable when needed.	Does the design keep important information visible, so that users do not have to memorize it? Does the design offer help in context?
Principle 7: Flexibility and Efficiency of Use	Shortcuts—hidden from novice users—may speed up the interaction for the expert user such that the design can cater to both inexperienced and experienced users. Allow users to tailor frequent actions.	Does the design provide accelerators such as keyboard shortcuts and touch gestures? Is content and functionality personalized or customized for individual users?
Principle 8: Esthetic and Minimalist Design	Interfaces should not contain information that is irrelevant or rarely needed. Every extra unit of information in an interface competes with the relevant units of information and diminishes their relative visibility.	Is the visual design and content focused on the essentials? Have all distracting, unnecessary elements been removed?
Principle 9: Help Users Recognize, Diagnose, and Recover From Errors	Error messages should be expressed in plain language (no error codes), precisely indicate the problem, and constructively suggest a solution.	Does the design use traditional error message visuals, such as bold, red text? Does the design offer a solution that solves the error immediately?
Principle 10: Help and Documentation	It is best if the system does not need any additional explanation. However, it may be necessary to provide documentation to help users understand how to complete their tasks.	Is help documentation easy to search? Is help provided in context right at the moment when the user requires it?

a Adapted from the “*Heuristic Evaluation Workbook*,” by K. Moran & K. Gordon, 2023, Nielsen Norman Group.

b Field labels are commonly used in forms to provide context for information input.

Consistent with Donald et al [29], the evaluators assessed the degree to which each of the 6 major functions from our digital platform complies with the 10 usability principles. They gave ratings of “−1 (does not comply),” “0 (partially complies),” or “1 (complies).” If an item scores “does not comply” or “partially complies” with a principle, the evaluator will be asked to note specific issues in not adhering to that principle and provide suggestions for fixing these problems. If the evaluator finds that the assessed principle does not apply to a specific function, they can select “N/A (not applicable).”

### Phase 3: Usability Testing

Usability testing involves observing how users interact with the platform to fulfill their needs; understanding their behaviors, preferences, operational strategies, and paths; and collecting feedback to identify existing interface problems and possibilities for improvement.

We designed a task for each of the 6 major functions of our digital platform, which a realistic user would typically use in their daily lives. Five dyads who participated in phase 1, with at least 1 month of experience using the platform, were invited to perform the designed tasks. As the DDEF emphasizes helping relationships, tasks were performed jointly by the dyads, with each assisting the other throughout the process. Researchers provided instructions on task execution and responded to participant queries but refrained from providing guidance to avoid influencing their behavior. Task design and testing followed usability testing guidelines from Moran [30] and Nielsen [31]. Task success, time spent, and the number of operational errors during execution were recorded for each task.

Upon completion of each task, whether successful or not, participants filled out a postscenario questionnaire (After-Scenario Questionnaire [ASQ]) consisting of three questions to assess the perceived (1) ease of the task, (2) time required, and (3) support received during execution (help from the manual) on a scale from 1 to 7 [32]. A higher average ASQ score indicates greater user satisfaction and perceived usability. After completing all tasks, participants also filled out the System Usability Scale (SUS), a 10-item questionnaire designed to evaluate the overall usability and learnability of the digital platform [33]. A SUS score ranges from 0 to 100, with 68 indicating marginally acceptable perceived usability by the user and suggesting the existence of areas for improvement [34]. See Multimedia Appendix 3 for the full usability testing instrument. The task scenario and instruction for each of the 6 functions are shown in Table 3.

**Table 3.** Usability testing task scenarios and instructions for tested functions.

Task scenario	Instruction	Tested function
Task 1: Here are the values of your blood pressure and blood test results from today’s visit: systolic blood pressure 125, diastolic blood pressure 78, heart rate 65, and creatinine (CREA) 1.4 mg/dL.	Please attempt to log the above values into our digital platform.	Record Values
Task 2: You want to know which stage of chronic kidney disease you are currently in. However, you found that your recent blood test report does not provide your “estimated glomerular filtration rate (eGFR)” value. You need to calculate your eGFR using “Creatinine (CREA)” (1.4 mg/dL), combined with your “gender” and “age.”	Please try to explore our digital platform and calculate your eGFR.	Health Education Information
Task 3: Here is the date and time of your next appointment: December 28, 2023, afternoon clinic; suggested check-in time 14:10.	Please update the above information on our digital platform and notify the researchers through the platform.	Reminder Settings
Task 4: You want to learn about the symptoms of “hypoglycemia.”	Please try to explore our digital platform and find the 7 possible symptoms of hypoglycemia.	Inquiry & Consultation
Task 5: Here is the content of your most recent meal: Half a bowl of white rice, 2 servings of bok choy, and 1 serving of chicken thigh.	Please record the above dietary information on our digital platform via photo or text note.	Kidney Health Mission
Task 6: Your partner (family member) has been trying hard to adjust their lifestyle recently. You want to know some ways to support their change.	Please try to explore our digital platform and write out support strategies corresponding to the following specific behaviors.	Kidney Support Teammate

### Data Analysis

Data were compiled and analyzed using Google Sheets. Categorical variables (eg, user responses in feedback forms) were summarized as frequencies and percentages. Continuous variables, such as ASQ and SUS scores, were reported using means and ranges. Task success rates, completion times, and error frequencies were recorded and descriptively summarized for each usability task. No inferential statistical analyses were conducted, as the study primarily aimed to assess usability outcomes through descriptive measures.

### Ethical Considerations

This study received approval from the institutional review board of National Cheng Kung University Hospital, Tainan, Taiwan (institutional review board no. B-ER-110-110). All participants were fully informed about the study’s purpose, procedures, their rights, and data protection measures. Participation was voluntary, and participants could withdraw at any time without consequence. Paper source data were collected and stored in locked cabinets on site at the institution. Deidentified research data were stored on the institution’s cloud servers and on password-protected personal computers, with access restricted to authorized study personnel only. All data will be retained until 2029 and then securely destroyed. Each participant received a signed copy of the informed consent form. Participants received a gift voucher of NT $200 (approximately US $6) as compensation each time they completed a study task.

## Results

### Phase 1: Iterative Development and Trial Use

After collaborating with health care providers and technical partners for 3 months to develop our digital platform for intervention, we officially launched the prototype on July 25, 2023. Our LINE OA, named “Kidney Lifestyle,” can be accessed by searching for LINE ID: @509kgajt. In addition, the extended app, named “Kidney Lifestyle App,” is currently available through a dedicated link.

#### LINE OA “Kidney Lifestyle” With API Plug-Ins

**Figure 2.**
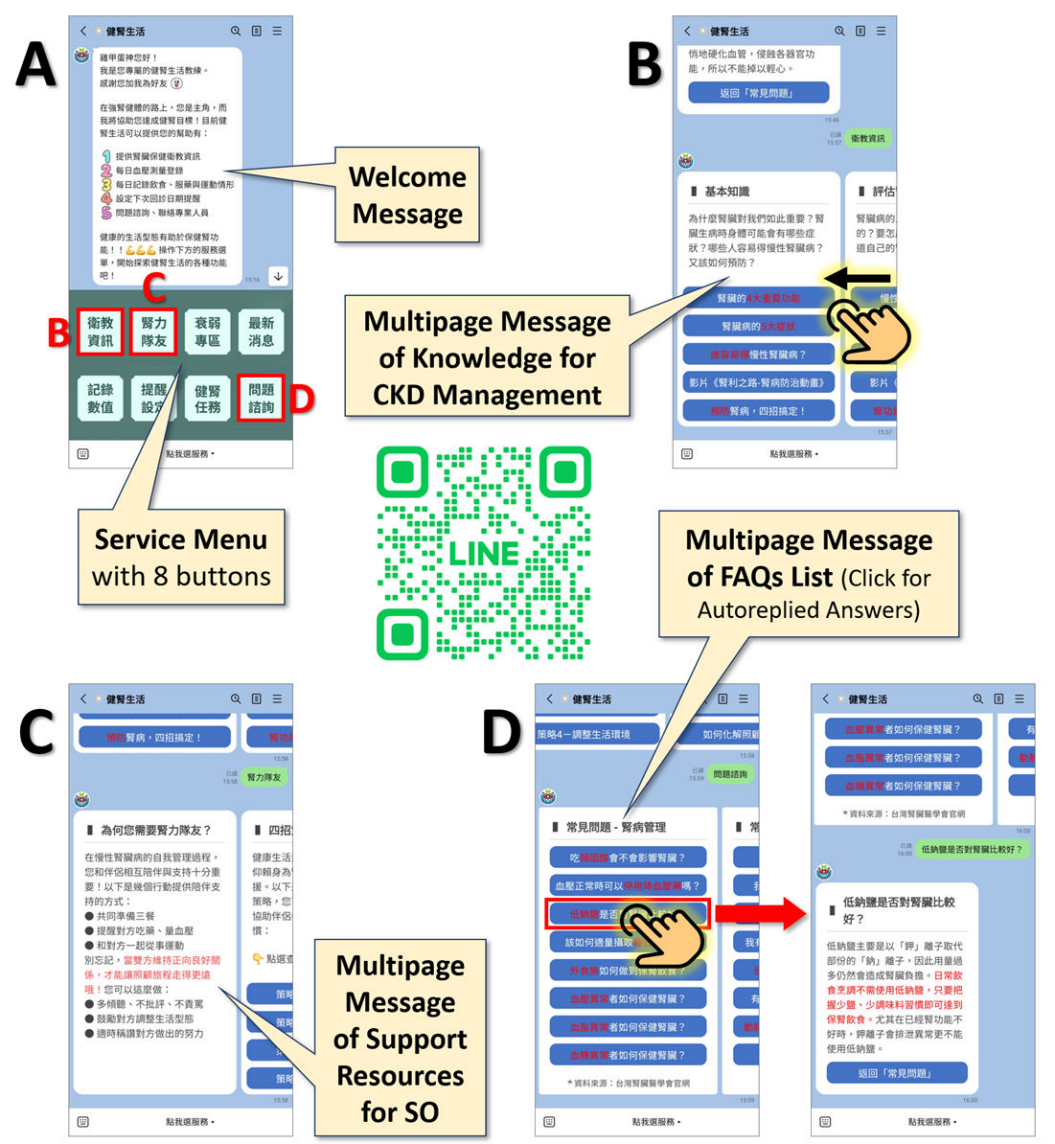
Interfaces of the LINE Official Account “Kidney Lifestyle” with a demonstration of user making inquiry about CKD management via automatic reply messages. The QR code provides access to “Kidney Lifestyle.” (A) Basic Interface, (B) Health Education Information, (C) Kidney Support Teammate, and (D) Inquiry & Consultation. CKD: chronic kidney disease; FAQs: frequently asked questions; SO: significant other.

Our LINE OA acts as the main hub for real-time interaction with users via text or voice chat. Moreover, by clicking buttons from the service menu (Figure 2A), users can access various resources for CKD management, retrieved or adapted from the official websites of *Taiwan Society of Nephrology* and *Taiwan Kidney Foundation*. The eight buttons featured on the service menu were introduced: (1) Health Education Information: providing basic kidney disease knowledge, evaluating kidney function, dietary and exercise guidelines, and so on (Figure 2B). (2) Kidney Support Teammate: multipage messages providing support resources for caregivers (SOs) (Figure 2C). (3) Frailty Section: providing health education information about frailty prevention and intervention. (4) Latest News: linking to the LINE OA’s post area, forwarding information about relevant hospital events. (5) Record Values: users can log self-measured physiological values, and the system records and generates reports for feedback. (6) Reminder Settings: users can log their next appointment dates, and reminders are scheduled through the AlleyPin Interactive backend. (7) Kidney Health Mission: encouraging daily completion of “Record Values” and “Kidney Health Diary” (recording daily diet, medication, and physical activity). (8) Inquiry & Consultation: providing answers to frequently asked questions for CKD management; a consultation channel is provided for further queries (Figure 2D).

#### The Extended App “Kidney Lifestyle App” (AppSheet)

**Figure 3.**
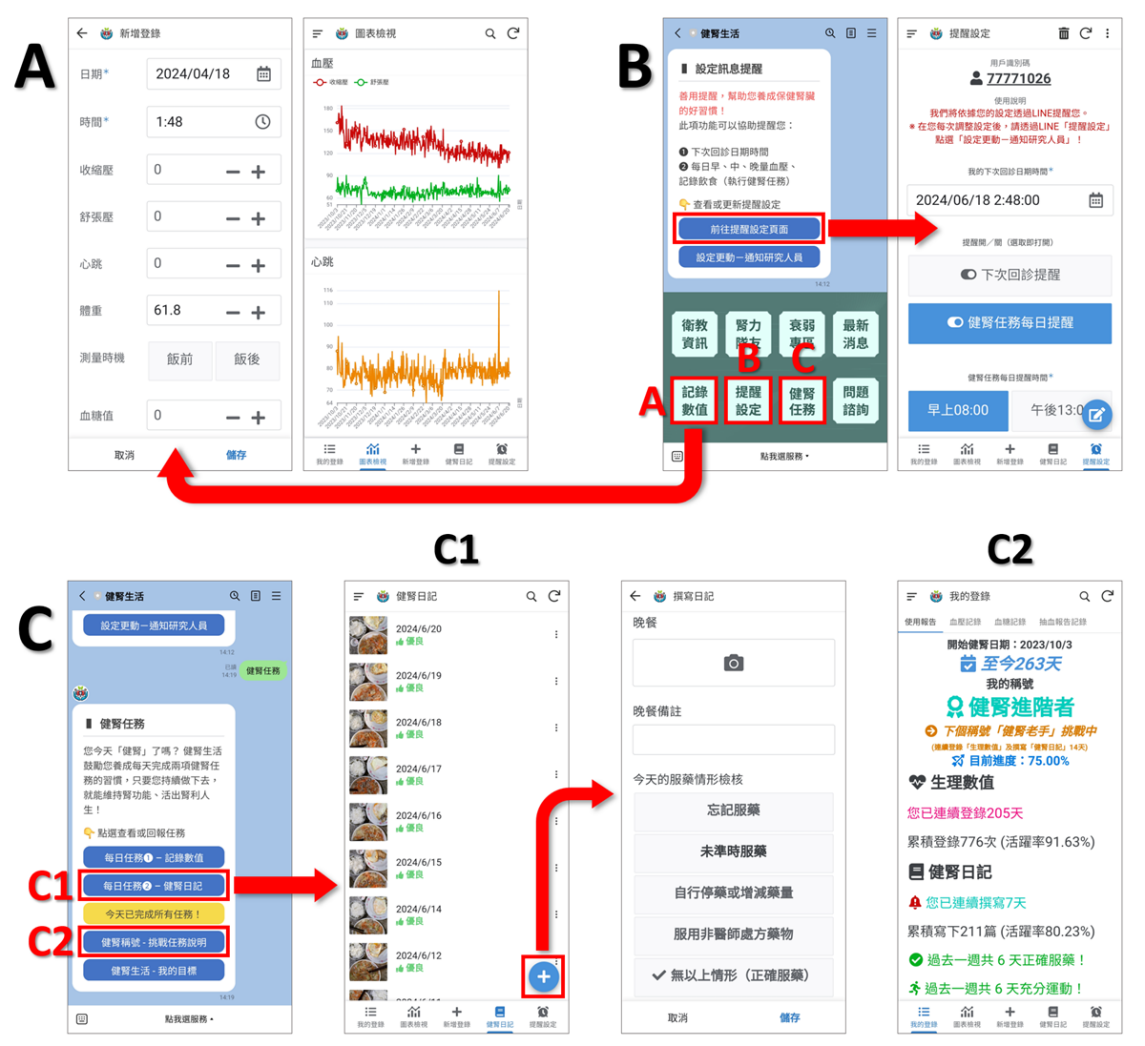
Interfaces of the extended app “Kidney Lifestyle App” and their connections with the LINE Official Account “Kidney Lifestyle” service menu. (A) The entry form of “Recording Values” and the chart viewing for self-measured physiological values, (B)“Reminder Settings” interface, and (C)“Kidney Health Mission,” directing to (C1) Kidney Health Diary, records of daily diet, medication, and physical activity, and to (C2) Usage Report, presenting daily tasks execution status and progress.

Our extended app mainly interconnects with the functions of “Record Values,” “Reminder Settings,” and “Kidney Health Mission” provided by our LINE OA with API plug-ins. Users can directly access the corresponding pages of the extended app by clicking on LINE OA service menu buttons, with the specific paths as follows: (1) Clicking “Record Values” on LINE OA service menu jumps to the “New Entry” page of the extended app, where users can log blood pressure, heart rate, and weight, and optionally enter blood sugar or blood test report data (Figure 3A). (2) Clicking “Reminder Settings” → “Go to Reminder Setting Page” on LINE OA service menu jumps to the extended app to view the next appointment date and time, which users can update manually (Figure 3B). (3) Clicking “Kidney Health Mission” on LINE OA service menu allows users to perform 2 “Daily Tasks,” jump to the extended app to log physiological values or write a kidney health diary (recording daily diet, medication, and physical activity) (Figure 3C1). Alternatively, they can click “Challenge Missions” → “View Title Challenge Progress” on the extended app to confirm task execution status (Figure 3C2).

In addition to the functionality accessible via LINE, the extended app interface also has a bottom-placed menu with five buttons (Figure 3), each briefly described as follows: (1) My Entries: viewing the “Usage Report” (the page redirected after clicking “View Title Challenge Progress” on LINE), “Physiological Value View,” and “Blood Test Record” to check entered values. (2) Chart View: visualizing self-entered physiological values in graphical form. (3) New Entry: same as clicking “Record Values” on LINE OA. (4) Kidney Health Diary: adding, viewing, or editing written kidney health diaries. (5) Reminder Settings: same as clicking “Go to Reminder Setting Page” on LINE OA.

The data entered by users on various pages of the extended app were synchronized with their device IDs and sent back to the corresponding Google Sheets worksheets. Through these worksheets, the team can view all user-entered data and identify them based on the device ID and a custom user identification code. Subsequently, security filters were set up, distinguishing by device ID, enabling individual users on the front end (ie, extended app interfaces) to access only their entered data.

With the completion of the extended app’s development and integration with the LINE OA, it can now fully use the “Digital Monitoring” strategy to allow both the research team and the users to track physiological values, daily diet, medication, and physical activity. For the application of the “Digital Analysis” strategy, we calculate user metrics such as accumulated entry counts, entry activity rates (EARs), and consecutive entry days. These serve as reference indicators for users’ lifestyle modification progress, enabling personalized interventions through the LINE OA.

#### Interventions to Promote Platform Use

Referring to the DDEF, interventions delivered through our integrated digital platform were designed and finalized through the following three processes:

1. Defined 4 specific lifestyle modification behaviors: physiological values monitoring, healthy diet, adequate physical activity, and proper medication.
2. Designed 2 daily tasks on the digital platform to execute these four specific behaviors:
  - Record Physiological Values: measure physiological values and log them through “Record Values” (multiple times allowed).
  - Write Kidney Health Diary: record daily diet (photographs or textual notes); check whether medication was taken correctly and, if not, select a reason; and check whether physical activity for more than 30 minutes was done and record the number of steps taken.

After consulting health care providers and observing practical scenarios, we confirmed the benefits of regular execution of the 2 daily tasks for both patients and health care providers. Recording physiological values generates easily readable reports, facilitating quick understanding of trends in physiological value changes. Writing a kidney health diary allows health care providers to confirm whether the patient’s meals align with the principles of a CKD diet, and it serves as a reminder for the patient to engage in physical activity and take medication correctly. Inappropriate medication check items reference the Chronic Kidney Disease Self-Care Scale [35] and the Elderly Medication Adherence Assessment [36], consolidated into 4 items: “missed medication,” “not taking medication on time,” “self-adjusted medication or dosage changes,” and “using nonprescribed medication.”

1. Designed strategies to promote the execution of daily tasks based on the principles of behavior change and processes of change listed in the DDEF, including:
  - Gamification Mode for Behavior Shaping: Different “Kidney Health Titles” are granted upon completing 2 daily tasks consecutively for 3 (*novice*), 7 (*intermediate*), 14 (*veteran*), 30 (*master*), and 60 (*grandmaster*) days. Users can view title challenge progress (in percentage) through the “Usage Report” page on the extended app.

To implement the gamification strategy, the team needed to calculate the number of consecutive days a user completed daily tasks from today backward. With the assistance of generative artificial intelligence, ChatGPT, for syntax writing, we used Google Apps Script to execute custom functions to solve this issue. Furthermore, the team further simulated data using RStudio (Posit Software, PBC) and attempted to interpret it using Item Response Theory to link the 2 indicators, “consecutive days” and “entry activity rate.” The calculation method for the EAR is as follows:


$$\text{Entry activity rate}=\frac{Days\;performing\;daily\;tasks}{Total\;observation\;days}\times 100\%$$

The EAR can be viewed as an “ability” indicator for users performing daily tasks. A higher EAR indicates a more regular execution of daily tasks. The team simulated different levels of EARs (from 0% to 100%, at intervals of 5%) for 180 observation days, with or without task execution, a total of 10,000 times. The frequency of achieving various Kidney Health Titles was calculated among these 10,000 simulations (Table 4).

**Table 4.** Occurrences of achieving different titles within 180 days based on various entry activity rates (simulation counts=10,000)^a^^,^^b^.

EAR^c^, %	Novice achieving counts	Intermediate achieving counts	Veteran achieving counts	Master achieving counts	Grandmaster achieving counts
0	0	0	0	0	0
5	211	0	0	0	0
10	1507	0	0	0	0
15	4040	1	0	0	0
20	6839	13	0	0	0
25	8873	64	0	0	0
30	9723	248	0	0	0
35	9954	698	0	0	0
40	9996	1590	2	0	0
45	10,000	2999	13	0	0
50	10,000	5003	48	0	0
55	10,000	7191	192	0	0
60	10,000	8852	502	0	0
65	10,000	9712	1275	0	0
70	10,000	9964	3001	17	0
75	10,000	9999	5629	82	0
80	10,000	10,000	8179	355	0
85	10,000	10,000	9746	1699	12
90	10,000	10,000	9994	5394	250
95	10,000	10,000	10,000	9541	3119
100	10,000	10,000	10,000	10,000	10,000

a EAR= (Number of days executing daily tasks/Total observation days) ×100%.

b Continuous execution days for each title: Kidney Health Novice—3 days; Kidney Health Intermediate—7 days; Kidney Health Veteran—14 days; Kidney Health Master—30 days; and Kidney Health Grandmaster—60 days.

c EAR: entry activity rate.

**Figure 4.**
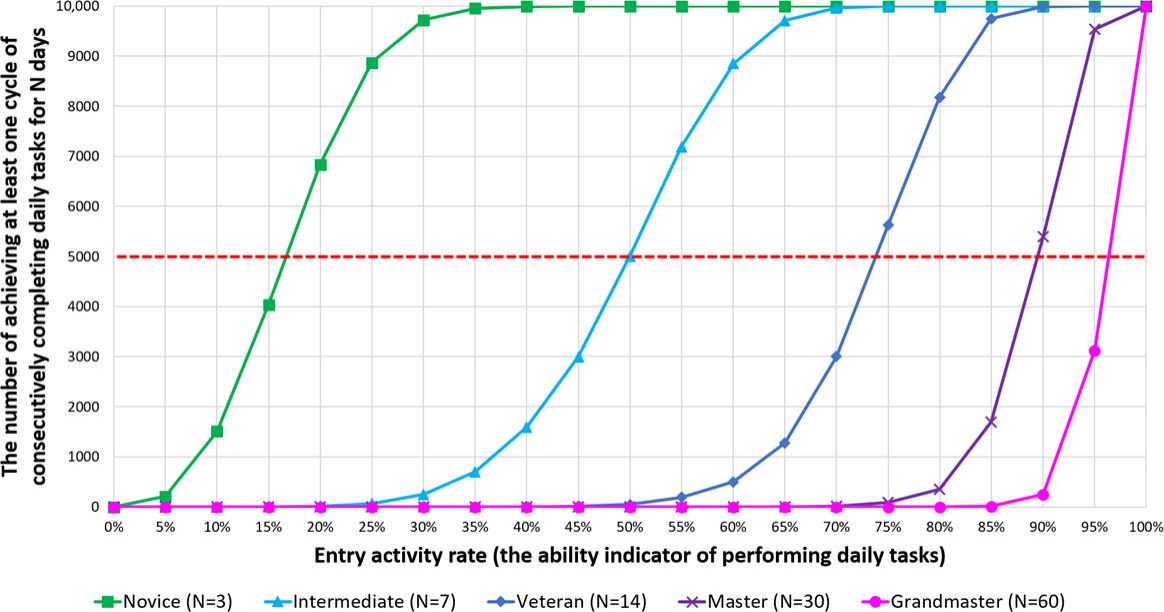
Item characteristic curves (ICCs) of achieving various kidney health titles within 180 days (simulation counts=10,000). According to Item Response Theory, with an achievement rate of 50% (ie, achieving 5000 times out of 10,000), the entry activity rate (EAR) corresponding to the ICC indicates the difficulty level to achieve that title. This simulation demonstrated the relationship between consecutively completing daily tasks for N days and its corresponding ability level, indicated by EAR. EAR = (Number of days executing daily tasks/Total observation days) × 100%. Continuous execution days for each title: Kidney Health Novice—3 days; Kidney Health Intermediate—7 days; Kidney Health Veteran—14 days; Kidney Health Master—30 days; and Kidney Health Grandmaster—60 days.

The simulation results provided item characteristic curve for each Kidney Health Title (Figure 4). These curves suggested that higher ability to perform daily tasks (indicated by a higher EAR) leads to a higher chance of achieving specific titles (indicated by more achievement instances). Using 5000 times of achievement as a baseline, its corresponding EAR on the item characteristic curve represents the difficulty level in achieving that specific Kidney Health Title. These results provide adequate rationality for the gamification strategy: assuming users in different stages of change have different EARs, this strategy sets appropriate challenges accordingly, aligning with our aim of providing corresponding interventions based on CKD dyads’ different motivational states.

- Reinforcement management: Upon completing 2 daily tasks, users receive encouragement messages through the LINE OA; when obtaining a Kidney Health Title, the team sends encouragement messages and title certificates via LINE and informs their SOs. It also informs the patient’s physician to provide face-to-face encouragement during the next visit (providing social reinforcement).
- Nudging through helping relationships: If a patient cannot complete tasks independently, the SO assists in recording. For patients in the early stages of change (eg, EAR below 50%) or those who have not performed tasks for more than 2 days, the system schedules LINE reminders via AlleyPin Interactive’s case tracking system or notifies SOs to assist in reminders. References are drawn from the resources in “Kidney Support Teammate,” providing assistance based on the patient’s lifestyle context.

#### Platform Trial Use to Optimize the Platform

After the initial prototype of our digital platform went live, 10 CKD dyads visiting nephrology clinics regularly at a university hospital were invited to participate in the trial use from August to October 2023. One of the SOs (no. 003S) was invited by the patient but did not decide to participate in the end, resulting in a total of 19 participants. All dyads completed the user feedback form and returned it during the next visit. Table 5 shows the basic demographics of the participants. The following sections separately analyzed (1) the LINE message activity, (2) usage of the extended app, and (3) dyadic feedback content.

**Table 5.** Basic information of participants in phase 1 platform trial use (n=19).

ID^a^	Sex	Age (years)	Education	Relations	Stage of change	Primary user^b^
001P and 001S				Spouse	Maintenance	P
001P	Male	67	College			
001S	Female	59	Junior college			
002P and 002S				Spouse	Maintenance	P
002P	Male	63	College			
002S	Female	62	Senior high			
003P and 003S				N/A	Preparation	P
003P	Male	64	College			
003S^c^	N/A^d^	N/A	N/A			
004P and 004S				Mother-son	Maintenance	S
004P	Female	81	Elementary			
004S	Male	53	College			
005P and 005S				Father-son	Maintenance	S
005P	Male	82	Illiterate			
005S	Male	56	Junior college			
006P and 006S				Mother-daughter	Preparation	P
006P	Female	37	College			
006S	Female	73	Junior college			
007P and 007S				Spouse	Maintenance	P
007P	Male	64	Junior high			
007S	Female	59	Elementary			
008P and 008S				Spouse	Contemplation	P
008P	Female	67	Junior college			
008S	Male	68	Junior high			
009P and 009S				Spouse	Action	P
009P	Male	52	College			
009S	Female	51	College			
010P and 010S				Mother-daughter	Maintenance	P
010P	Female	70	Senior high			
010S	Female	42	Junior college			

a ID marked with P denotes patients; S denotes significant others.

b Primary user refers to the one using the app for daily tasks, primarily patients.

c 003S was invited by 003P to participate in the study but eventually chose not to.

d N/A: not applicable.

##### LINE Message Activity

Based on data from the LINE OA backend, a total of 849 messages were sent during the initial 28 days (October 30 to November 26, 2023) after participant recruitment completion (as shown in Table 6), averaging approximately 30 messages per day. This indicates continuous interaction and usage.

**Table 6.** Number of messages sent via LINE Official Account and application programming interface module in the initial 28 days after participant recruitment completion^a^.

Date	LINE Official Account			API^b^ module		Daily total
	Auto Response	Welcome	Manual Chat	Push^c^	Reply^d^	
10/30	1	5	2	12	55	75
10/31	0	0	0	4	20	24
11/1	0	1	0	15	22	38
11/2	0	3	6	17	46	72
11/3	0	0	7	13	17	37
11/4	0	0	4	11	6	21
11/5	0	1	0	9	11	21
11/6	0	0	0	12	22	34
11/7	0	0	1	11	2	14
11/8	0	1	1	18	11	31
11/9	0	2	2	15	13	32
11/10	0	0	2	11	13	26
11/11	0	0	0	9	14	23
11/12	0	0	1	11	17	29
11/13	0	0	1	9	8	18
11/14	0	2	3	18	16	39
11/15	0	1	1	15	9	26
11/16	0	1	3	15	21	40
11/17	0	0	3	11	19	33
11/18	0	0	2	11	6	19
11/19	0	0	1	9	3	13
11/20	0	0	2	15	12	29
11/21	0	1	3	11	12	27
11/22	0	1	3	18	45	67
11/23	0	0	1	10	5	16
11/24	0	1	1	11	2	15
11/25	0	0	2	9	5	16
11/26	0	0	1	12	1	14
Total	1	20	53	342	433	849

a Data obtained from LINE Official Account Manager.

b API: application programming interface.

c Push messages sent to remind users of follow-up visits and daily tasks execution.

d Messages returned when clicking service menu layers or push message buttons.

Notably, the proportion of messages sent through API plug-in modules is extremely high: there were 342 Push messages, accounting for approximately 40% (342/849) of all messages, used for reminders on returning for checkups or daily task reminders and regular automatic care. There were also 433 Reply messages, constituting around 51% (433/849) of all messages, automatically sent by the system when users clicked buttons in the service menu or Push message prompts. In comparison, manually sent messages amounted to only 53, which is around 6% (53/849) of the total, primarily aimed at assisting users with operational issues or scheduling research-related appointments. These data demonstrate that our LINE OA with API plug-in modules shows a high degree of automation and convenience, allowing remote and real-time intervention, significantly reducing manpower costs, and reserving resources for handling more complex and specific user queries.

##### Usage of the Extended App

Upon observing the data on daily task execution by the primary users among the 10 dyads on the extended app (as shown in Table 7), since the start of enrollment on August 29, 2023, until 2:30 PM on April 29, 2024, a total of 2402 sets of physiological values have been logged, and 1079 kidney health diaries have been uploaded. Notably, it was found that the average EAR for logging physiological values (63.70%) was higher than that for writing kidney health diaries (50.74%). This difference might be due to the ease of recording physiological values and the immediate feedback obtained through trend chart viewing.

**Table 7.** Phase 1 primary users’ usage of extended app for daily tasks (n=10)^a^.

ID	Enrollment date (YY/M/D)	Days	Physiological values		Kidney health diary		Highest kidney health title^d^
			Counts	Activity rate, %^b^	Counts	Activity rate, %^c^	
001P	23/8/29	245	530	95.92	232	94.69	Master
002P	23/9/18	225	438	100.00	209	92.89	Master
003P	23/9/21	222	164	62.61	133	59.91	Intermediate
004S	23/10/2	211	2	0.95	0	0.00	None
005S	23/10/3	210	579	89.52	168	80.00	Master
006P	23/10/17	196	100	51.02	7	3.57	Novice
007P	23/10/19	194	125	63.92	25	12.89	Intermediate
008P	23/10/23	190	0	0.00	0	0.00	None
009P	23/10/23	190	307	90.53	161	84.74	Master
010P	23/10/30	183	157	82.51	144	78.69	Master
Total (Average)		206.6	2402	63.70	1079	50.74	N/A^e^

a This table is updated until April 29, 2024, at 2:30 PM.

b Activity rate for physiological values = (Days logged/Days enrolled) × 100%.

c Activity rate for kidney health diary = (Days written/Days enrolled) × 100%.

d Continuous execution days for each Kidney Health Title: novice—3 days; intermediate—7 days; veteran—14 days; and master—30 days.

e N/A: not applicable.

Eight users had an activity rate higher than 50% for logging physiological values, and among them, 6 also had an activity rate higher than 50% for writing kidney health diaries, indicating these users have a moderate to high level of acceptance and capability in performing daily tasks on the extended app. Regarding the challenges for Kidney Health Titles: 8 out of 10 users achieved consecutive logging of physiological values and writing kidney health diaries for at least 3 days (granted the “Kidney Novice” title) during the observation period; among the previously mentioned highly active 6 users, they achieved challenges of logging physiological values and writing kidney health diaries consecutively for 7 days (*intermediate*) or even 30 days (*master*). This demonstrates that higher activity levels correlate with a higher chance of long-term adoption of a healthy lifestyle, aligning well with the conclusions drawn from the simulation study.

##### Dyadic Feedback Content

Table 8 summarized the average ratings for each of the 6 major functions of our integrated platform, as provided by the 10 dyads in their user feedback forms. Generally, more than 80% of the dyads (more than 8) perceived each function to have a clear interface and to be easy to use. Similarly, most of the dyads (more than 80%) found each function acceptable and expressed their intention to continue usage. The average satisfaction rating for each function ranged from 3.5 to 4.3, with an overall rating of 4.1 for the platform as a whole.

**Table 8.** Phase 1 dyadic trial use feedback form rating summary (N=10)^a^.

Functions of the digital platform	Assessed elements				
	#N-1: Interface clarity	#N-2: Ease of operation	#N-3: Acceptability	#N-4, 7‐2: Intention to continue usage	#N-5, 7‐2: Satisfaction rating (from 1 to 5)
Health Education Information	90%	90%	90%	90%	3.9
Kidney Support Teammate	90%	100%	90%	90%	3.5
Record Values	90%	80%	90%	80%	4.3
Reminder Settings	100%	90%	90%	90%	4.1
Kidney Health Mission	80%	90%	80%	80%	3.8
Inquiry & Consultation	100%	90%	80%	90%	3.7
Average	91.67%	90%	86.67%	86.67%	3.9
Overall platform evaluation	N/A^b^	N/A	N/A	90%	4.1

a The percentages in this table reflect the proportion of participant dyads who answered “Yes” to the corresponding Yes/No questions in the user feedback form.

b N/A: not applicable.

Qualitative feedback on each function, gathered through user feedback forms and brief interviews, is organized according to its positive and negative aspects:

1. Health Education Information (LINE): Dyads generally appreciated this function for its simplicity, clarity, and informative nature, noting that “it provided access to various, accurate CKD-related information at any time” (no. 005). However, one dyad (no. 010) suggested that “the font size should be enlarged, and the navigation of this function needs simplification to avoid getting lost.*”*
2. Kidney Support Teammate (LINE): The majority of dyads found this function to be beneficial, appreciating its provision of “mutual support, encouragement, reminders, and detailed knowledge” (nos. 001 and 002). However, 2 dyads expressed concerns regarding its usage, with one noting “not using this function” (no. 005) and the other mentioning that “the colors are too vibrant, making it difficult to grasp key points, and navigating within the function could lead to getting lost” (no. 007).
3. Record Values (LINE+ App extension): Overall, dyads expressed satisfaction with this function, noting its advantages in “replacing traditional paper records, convenience, ease of use, and the ability to track data” (nos. 001, 002, and 009). Specifically, 1 dyad (no. 005) appreciated the support for chart view upon logging in data, facilitating easy tracking. However, 1 dyad (no. 010) mentioned dissatisfaction with the small font size and confusion regarding certain actions due to unclear wording.
4. Reminder Settings (LINE+ App extension): Dyads expressed overall satisfaction with this function, particularly highlighting its capability to provide advance notifications for upcoming clinical appointments. This function was deemed convenient and provided peace of mind by several dyads (no. 001, 005, and 009), eliminating the need to remember appointment dates. No negative feedback was received about this function.
5. Kidney Health Mission (LINE+ App extension): Overall, dyads who appreciated this function found its operation to be straightforward. Some dyads specifically noted its convenience for recording daily diet by uploading a photograph (no. 005) and its provision of daily data for tracking (no. 002). Conversely, 1 dyad found this function “troublesome, causing psychological pressure,” and despite its good intent, felt “it lacked feedback from healthcare providers” (no. 001). Another dyad expressed inconvenience due to the small font size and difficulties logging in data (no. 010).
6. Inquiry & Consultation (LINE): Dyads provided positive feedback regarding the helpfulness of this function, particularly emphasizing its ability to provide answers to various relevant questions (nos. 002, 005, and 009). One dyad noted that “for new CKD patients, this function could serve as a substitute for in-person health education; however, for experienced patients, this function may seem unnecessary as they are already familiar with the answers” (no. 001). Another dyad commented on the potential for getting lost while navigating through the content of this function (no. 007).

Regarding the overall evaluation of the digital platform, neither dyad had previously used other apps or websites for managing CKD. Almost all of the dyads (90%) expressed willingness to continue using it to aid in managing their CKD. Upon further in-depth interviews with researchers about dyadic feedback, all agreed that the digital platform’s functions were comprehensive, with no suggestions for additional functions. The first patient (no. 001P) mentioned that using the platform provided a “routine” after retirement. His SO (no. 001S) also mentioned the lack of diverse social activities after retirement for the patient and expressed interest in participating in hospital support groups to share their experiences with other CKD dyads.

### Phase 2: Heuristic Evaluation

By early November 2023, the team collected responses from 5 evaluators who participated in the heuristic evaluation. The evaluators’ basic information is shown in Table 9.

**Table 9.** Basic information of phase 2 heuristic evaluation participants (N=5).

No.	Sex	Age (years)	Education	Background	Current occupation
1	Female	55	PhD	Nursing	Professor
2	Female	42	PhD	Health care science	Associate professor
3	Female	45	PhD	Nursing	Assistant professor
4	Female	54	PhD	Nursing	Assistant professor
5	Male	28	Master’s degree	Electrical engineering	Engineer

#### General Compliance Rating

Their scores regarding the compliance of various functions with usability principles were tallied, averaged, and summarized in Table 10. On average, the compliance scores for each principle ranged between 0 (*partially complies*) and 1 (*complies*). The functions’ average compliance with the 10 usability principles ranged from 89% to 93%. This suggests that the evaluators generally perceived that the digital platform’s various functions mostly complied with the usability principles.

**Table 10.** Phase 2 heuristic evaluation compliance rating summary (N=5)^a^.

Degree of compliance with each usability principle	Functions of the digital platform					
	Health Education Information, %	Kidney Support Teammate, %	Record Values, %	Reminder Settings, %	Kidney Health Mission, %	Inquiry & Consultation, %
Principle 1	60	80	100	80	80	100
Principle 2	100	100	100	100	80	100
Principle 3	100	80	80	60	100	75
Principle 4	100	100	100	100	100	100
Principle 5	100	100	100	100	100	100
Principle 6	80	80	100	100	100	75
Principle 7	80	80	100	60	100	80
Principle 8	100	100	100	100	100	100
Principle 9	100	100	33.33	100	33.33	100
Principle 10	100	100	100	100	100	100
Overall result	92	92	91.33	90	89.33	93

a Compliance percentages represent the sum of evaluator ratings (*−*1, 0, or 1) for each function’s compliance with each usability principle, divided by the number of valid responses (ie, excluding “N/A [not applicable]” selections), and then multiplied by 100%.

#### Recommendations for Improvement

The breakdown of issues raised by evaluators about each function’s incompliance with certain principles and their recommendations is as follows:

1. Health Education Information

- Principle 1 (60% compliance): Evaluator 1 found that “clicking into the five stages of CKD and lab test reports didn’t lead to corresponding content.” Evaluator 5 suggested “transforming health education information into daily articles and actively pushing them to users with thematic context.”
- Principle 6 (80% compliance): Evaluator 2 felt that the design here might cause information loss or be easily forgotten after closing the linked web page and suggested implementing a memory function for highly clicked queries.
- Principle 7 (80% compliance): Evaluator 2 believed that this function lacked personal flexibility and suggested establishing personalized zones based on click rates to access or push commonly used information.

2. Kidney Support Teammate

- Principle 1 (80% compliance): Evaluator 5 felt that while the information was comprehensive, the incentive for users to actively read this information was slightly weak and suggested recommending correct concepts based on user records.
- Principle 3 (80% compliance): Evaluator 2 noticed that once clicked, the buttons for some information, returning to the previous message is not supported, requiring scrolling or clicking on the service menu again, recommending immediate display of original options after dialogue box content appears.
- Principle 6 (80% compliance): Same as evaluator 2’s feedback in (1) “principle 6 (80% compliance).”
- Principle 7 (80% compliance): Same as evaluator 2’s feedback in (1) “principle 7 (80% compliance).”

3. Record Values

- Principle 3 (80% compliance): Evaluator 1 noted that after entering values, the entire app needed closing to return to the main screen and suggested adjusting this.
- Principle 9 (33% compliance): Evaluator 5 noticed that there were no notifications for abnormal physiological values and suggested providing automatic or manual messages based on professional or objective facts for abnormal values.

4. Reminder Settings

- Principle 1 (80% compliance): No specific issues or suggestions were mentioned by the evaluator.
- Principle 3 (60% compliance): Evaluator 5 discovered that alterations in the reminder function required notification to researchers and suggested allowing users to freely set daily or appointment reminders, similar to setting alarms on a phone.
- Principle 7 (60% compliance): Same as evaluator 5’s feedback in (4) “principle 3 (60% compliance).”

5. Kidney Health Mission

- Principle 1 (80% compliance): Evaluator 5 felt that the current daily diet recording was based on self-assessment without professional guidance and suggested having professionals review information and provide guidance.
- Principle 2 (80% compliance): Same as evaluator 5’s feedback in (5) “principle 1 (80% compliance).”
- Principle 9 (33% compliance): Same as evaluator 5’s feedback in (3) “principle 9 (33% compliance).”

6. Inquiry & Consultation

- Principle 3 (75% compliance): Same as Evaluator 2’s feedback in (2) “principle 3 (80% compliance).”
- Principle 6 (75% compliance): Same as evaluator 2’s feedback in (1) “principle 6 (80% compliance).”
- Principle 7 (80% compliance): Same as evaluator 2’s feedback in (1) “principle 7 (80% compliance).”

Overall, based on the evaluation scores and feedback from evaluators on each function of the digital platform concerning various principles, this phase successfully acquired specific recommendations for improving the platform’s usability. These suggestions can generally be categorized into three aspects: (1) enhancing user operation convenience, such as adding a return option in Health Education Information, Kidney Support Teammate, and Inquiry & Consultation, reducing repetitive operation hassles; (2) pushing more relevant and engaging information, for instance, pushing corresponding educational content based on user records; and (3) enhancing interactivity and feedback of functions, such as providing personalized memory functions for highly clicked content, warning after inputting abnormal physiological values, or providing feedback from professionals. Results of the heuristic evaluation have guided the modification of the digital platform for the next iteration to enhance user experience.

### Phase 3: Usability Testing

Following the design of tasks, the selection of participants for phase 3 involved convenience sampling by inviting 5 dyads who participated in phase 1 trial use during their next clinic visits. Eventually, dyad numbers 001, 002, 005, 007, and 009 agreed to take part in the testing. The results of their average performance on our designed tasks and their average ASQ ratings are summarized in Table 11.

**Table 11.** Phase 3 usability testing task performance and rating summary (N=5).

	Functions of the digital platform					
	Health Education Information	Kidney Support Teammate	Record Values	Reminder Settings	Kidney Health Mission	Inquiry & Consultation
Task success^a^	60%	80%	100%	100%	100%	80%
Task time (mm:ss)	04:23	05:16	01:06	01:52	01:04	02:55
Average error times (range)	1 (0‐2)	1.4 (1-2)	0.2 (0‐1)	0.8 (0‐1)	0	1.2 (0‐2)
ASQ: ease of use^b^	3.6	4	6.2	5.4	6	4.2
ASQ: time required	3.4	4.2	6.4	5.2	6.2	4.2
ASQ: help received	3.8	3.8	6	4.2	5.2	3.6
Average ASQ score	3.6	4	6.2	4.9	5.8	4

a Task success = (Number of dyads successfully completed the task/N) × 100%.

b ASQ: After-Scenario Questionnaire. The score of ASQ ranges from 1 to 7, with higher score indicating higher level of satisfaction.

#### Task Performance and ASQ

This section describes the designed task scenario for each function and summarizes participants’ general task performance as well as their ASQ ratings:

1. Health Education Information: The task scenario reflected the need for patients with CKD to calculate their estimated glomerular filtration rate (eGFR) independently. To accomplish this task, users can navigate through the Health Education Information section in our LINE OA to find a link to a web page with an eGFR calculator. Results indicated that 3 out of 5 dyads completed this task successfully (success rate 60%), with an average completion time of approximately 4.5 minutes. Operational errors were observed with numbers 002, 005, and 009, who navigated to the wrong sections, causing them additional time to locate the eGFR calculator. Another error occurred when number 007 could not find the box to enter the value of creatinine (CREA), as the enlarged font size setting obscured a clear presentation of the calculator elements on the smartphone. The average ASQ score for this task is 3.6, which is the lowest score among all tasks, indicating that dyads were the least satisfied with the ease and the time required for this task. They also perceived minimal help from the user manual.

2. Kidney Support Teammate: The task scenario involved an SO finding effective ways to support a patient who is attempting lifestyle modification. The Kidney Support Teammate in our LINE OA provides such information for SOs to implement supportive strategies. The task success rate is 80%, with only 1 dyad failing to complete this task. The average completion time exceeded 5 minutes, which is the longest among all tasks. All dyads took time to navigate different sections before they located the target information for this task. Dyads numbers 005 and 009 experienced uncertainty as they browsed through the correct section but did not recognize the target information. The average ASQ score is 4, indicating a moderate but relatively low level of user satisfaction compared with the other tasks.

3. Record Values: The task scenario reflects the need for patients with CKD to monitor their own physiological data. Completing this task requires the user to click “Record Values” on the service menu of our LINE OA or click “New Entry” on the extended app and input the numbers. The task success rate is 100%, with an average completion time of only 1 minute. Dyad number 007 encountered initial difficulty when trying to input the CREA value. The average ASQ score is 6.2, which is the highest among all tasks. These results suggested that this function is user-friendly and intuitive, leading to high user satisfaction.

4. Reminder Settings: The task scenario arises when patients with CKD wish to set a reminder for their upcoming clinical appointment. Dyads will need to input the date and time of their next appointment using the extended app and notify the research team via LINE OA to set an automatic message reminder. The task success rate is 100%, with an average completion time of around 2 minutes. Dyads numbers 002 and 007 seemed unfamiliar with this function as they clicked “New Entry” on the extended app, which redirected them to the wrong page. The average ASQ score is 4.9, indicating a moderate satisfaction level with this function.

5. Kidney Health Mission: The task scenario revolves around the essential practice of tracking daily diet, a key aspect of managing CKD. Users can access this task by clicking “Kidney Health Mission” on the service menu of our LINE OA or selecting “Kidney Health Diary” on the extended app to upload a photograph or text note regarding their diet. The task success rate is 100%, with an average completion time of just 1 minute. All dyads completed the task without encountering any operational errors, indicating that the design of this function is sufficiently intuitive. The average ASQ score is 5.8, demonstrating a relatively high level of satisfaction compared with the other tasks.

6. Inquiry & Consultation: This task scenario arises when patients with CKD encounter health-related inquiries at home without access to a health care professional. Users can explore the frequently asked question section in our LINE OA to seek answers to their questions. For instance, this task involves users wanting to learn about the symptoms of hypoglycemia (low blood sugar) using our platform. The task success rate is 80%, with dyad number 002 failing to complete the task. On average, it takes around 3 minutes to complete the task, as all dyads navigate through different sections of our LINE OA to find the desired information. Errors occurred when all dyads, except number 001, attempted to locate the answer in the Health Education Information section. The average ASQ score is 4, indicating a moderate but relatively low level of user satisfaction, highlighting areas for usability improvement.

#### Overall Platform Usability

The SUS scores provided by the 5 dyads range from 57.5 to 87.5, with an average score of 67.5, just below the industry standard threshold of 68. This indicates that users found the platform to be marginally acceptable in terms of usability, indicating areas for improvement. In combination with the performance and ASQ ratings of the 6 tasks, it was observed that the 3 tasks involving operations on the extended app (ie, Record Values, Reminder Settings, and Kidney Health Mission) all achieved a 100% success rate, with shorter completion times, fewer operational errors, and higher satisfaction (mean average ASQ score: 5.64 vs 3.87 for the other 3 tasks). Conversely, tasks with lower performance and ASQ scores (ie, Health Education Information, Kidney Support Teammate, and Inquiry & Consultation) all involved finding information within LINE OA, suggesting that underlying significant usability issues in this area may have contributed to the low overall platform SUS score.

## Discussion

### Principal Results

In this study, we demonstrated that through an instant messaging app, our digital platform aimed at empowering lifestyle modification for CKD dyads holds promise for its cost-effectiveness, high user engagement, and potential clinical practicality. Key insights emerged from the 3-phase development cycle are as follows.

First, the application of Agile methods facilitated a flexible and efficient process in developing our digital platform. Notably, the platform design and deployment were accomplished within a remarkably short time frame of 3 months, with the ability to accommodate modifications, typically within a week. Such adaptability of our platform ensured responsiveness to user needs throughout the development cycle.

Second, guided by the DDEF, this study introduced 2 quantifiable daily self-management tasks—logging physiological values and maintaining a kidney health diary—to address the multifaceted nature of CKD management. These tasks, coupled with behavior change techniques, aimed to promote sustained user engagement. The EAR of these tasks emerged as a simple, innovative, and promising indicator of users’ lifestyle modification abilities, which could facilitate more precise tailored interventions based on different user activity levels.

Third, comprehensive evaluation across 3 phases revealed high user acceptance and satisfaction with our digital platform. However, usability challenges, particularly concerning information layout within the LINE OA, were identified. The absence of CKD dyad involvement during the initial prototype development hindered the alignment of the content of LINE OA service menu with varying user mental models, potentially causing users to struggle with navigation [37]. This finding underscores the importance of considering user expectations early in the development cycle to ensure optimal usability.

### Comparison With Prior Work

Previous DHIs for CKD management have adopted various approaches, including a mobile app [15], a web-based platform [17], a chatbot [21], and the combination of a mobile app and wearable devices with instant messaging support [20]. These interventions have demonstrated potential in supporting CKD health education and self-management. However, challenges were faced such as limited evidence of sustained use—many reported low [15] or unclear usage rates [17,20,21]—and participant samples skewed toward younger, tech-savvy patients (mean age around 50 years), due to the necessity of internet access and smartphone proficiency. Moreover, prior systems typically focused on a single aspect of care (education or tracking) and rarely involved patients’ family members, potentially excluding older patients who require caregiver support. In contrast, our LINE-based digital dyadic empowerment platform addresses these gaps through the following strengths mentioned in the subsections.

#### Diverse Participant Backgrounds

Our study encompassed participants with diverse backgrounds, ranging from health care providers (including nephrologists and nurses) within the institution involved in phase 1 system development, to patients with CKD and SOs engaged in both phase 1 platform trial use and phase 3 usability testing. Health care and electrical engineering experts from external institutions were involved in phase 2 heuristic evaluation. Of these participants, 41.7% (10/24) were male, spanning ages from 28 to 82 years, and educational attainment ranging from elementary school to doctoral degrees. Such diversity maximized the input from all potential stakeholders who could use or benefit from our digital platform. We ensured that various perspectives were considered throughout the development cycle, thereby optimizing platform usability while accommodating user differences.

Notably, the design of including SOs with patients with CKD in our study was pivotal. It not only enriched the diversity of perspectives but also prevented the exclusion of patients who might face challenges participating independently, such as those from older age groups or with lower educational levels, potentially constituting digitally disadvantaged groups. This inclusive approach, exemplified by dyads numbers 004 and 005 (Table 5), served to mitigate selection bias and promote health equity at the digital resource level.

#### A Scalable and Practical Digital Platform

In a 2023 internet survey conducted in Taiwan, it was found that 98.51% of internet users use instant messaging tools, with LINE holding a dominant market share of 91.61% [38]. Our digital platform operates within Taiwan’s most prevalent instant messaging app, significantly improving its accessibility and ubiquity. This integration closely aligns CKD self-management with the most frequent daily use of digital technology. Data extracted from the backend of the LINE OA further revealed consistent user engagement with our digital platform, with more than 90% of interactions being automatic Push or Reply messages (Table 6). This underscores the high automation and low-cost model of our digital platform, demonstrating its potential for scalable application in medical settings.

**Figure 5.**
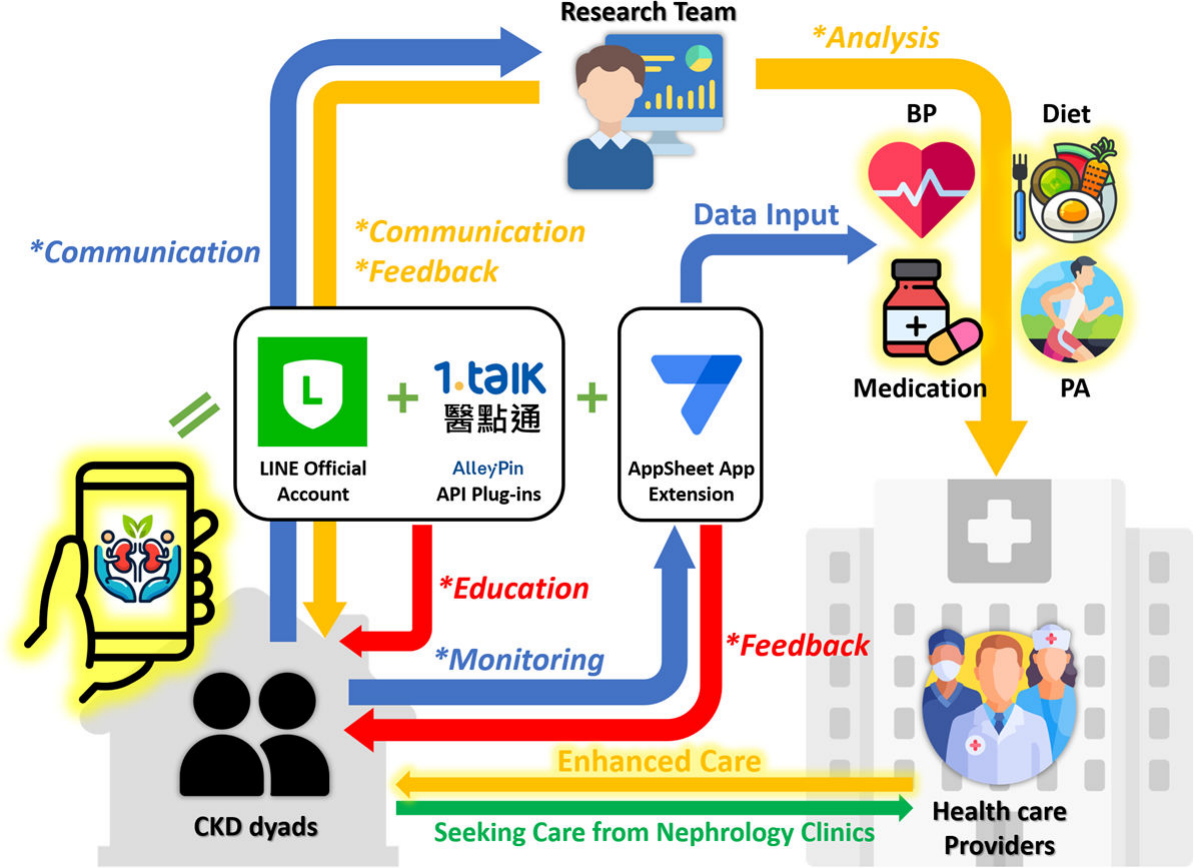
The schematic diagram of the LINE Official Account “Kidney Lifestyle” and the extended app in enhancing CKD care. The arrows marked with an asterisk (*) represent the practical application of the 5 digital strategies mentioned in the Digital Dyadic Empowerment Framework. Blue arrows represent the active engagement from CKD dyads. Orange arrows denote service flows involving human support, while red arrows denote service flows with full automation. BP: blood pressure; CKD: chronic kidney disease; PA: physical activity.

Figure 5 further illustrates the interactive relationship between CKD dyads, the research team, and health care providers, elucidating how our digital platform can enhance CKD care in medical settings. The green arrow at the bottom represents patients with CKD and their SOs seeking routine care at the hospital. Blue arrows depict CKD dyads using LINE OA, either through encouragement or voluntary interaction, to stay connected with the research team (*communication* strategy); or using the extended app to log home-based health records, including blood pressure, diet, medication, and physical activity (*monitoring* strategy). Red arrows signify the fully automated services offered by our digital platform, such as providing health education information through LINE OA Push or Reply messages (*education* strategy); or accessing physiological trend charts and usage reports via the extended app (*feedback* strategy). Orange arrows indicate the interventions that the research team can deliver through the digital platform, such as maintaining contact with CKD dyads (*communication* strategy); or answering CKD dyads’ queries and sending encouraging messages (*feedback* strategy) via LINE OA. Finally, the research team can further analyze and aggregate home-based self-monitoring records logged by CKD dyads through the extended app (*analysis* strategy) and provide reports to health care providers. This additional information will furnish health care providers with supplementary insights during CKD dyads’ visits, aiding in treatment recommendations to enhance the quality of CKD care.

#### Empirical Evidence for Sustained User Engagement

This study provides empirical evidence of high user acceptance and satisfaction with our digital platform, as indicated by self-reported satisfaction ratings and intentions to continue usage during phase 1 trial use, as well as by the ASQ scores obtained from phase 3 usability testing. While self-reported ratings could potentially be inflated by demand characteristics [39,40], the robustness of these findings is further supported by users’ consistent execution of the 2 daily self-management tasks. As depicted in Table 7, more than half of the 10 trial users maintained an EAR from 50% to 100% for the 2 daily tasks for more than 6 months. Notably, 5 users had a record of executing the 2 daily tasks consecutively for 30 days or more, demonstrating their ability to sustain regular usage. These findings imply their persistent commitment to using our digital platform for managing CKD, even after they completed the study. Such ongoing engagement serves as compelling evidence of our digital platform’s potential as a highly acceptable digital health solution, empowering CKD dyads in the process of lifestyle modification.

### Limitations

#### Potential Selection Bias Induced by Digital Divides

Despite the wide availability of our digital platform on LINE OA and the inclusive dyadic design, this study might still have suffered from selection bias due to digital divides between those with access to our platform and those without [41]. During phase 1 participant recruitment, we encountered instances where some CKD dyads, particularly spouses, reported lacking access to smartphones or the internet. Conversely, some dyads had smartphones with internet access but lacked diverse digital skills or did not have LINE installed on their phones. All these factors could impede the implementation of our study and raise concerns over health inequity. Although the extent of these digital divides was not thoroughly investigated within the scope of this study, their existence warrants careful consideration in our future work to prevent selection bias favoring participants with higher digital proficiency.

For participants facing difficulties in using our platform, personalized assistance will be provided through interactive learning sessions and the user manual, reducing the likelihood of their attrition from the study. Furthermore, future research could investigate the prevalence and characteristics of digitally disadvantaged groups within the CKD population. Assessing the severity and underlying causes of digital divides may help mitigate their impact on the implementation of our DHI. Alternatively, programs empowering lifestyle modification tailored to CKD dyads who are not internet or smartphone users could be developed.

#### Usability Issues With LINE OA Navigation

Despite continuous optimization, the SUS scores obtained from phase 3 usability testing indicate that the usability of our digital platform remains marginally acceptable. Particularly, significant usability issues were identified within the information layout of the service menu on LINE OA. During phase 1 trial use, dyads reported difficulties navigating sections including Health Education Information, Kidney Support Teammate, and Inquiry & Consultation, often getting lost. Similarly, task performance and user satisfaction related to these sections were relatively low in phase 3 usability testing. However, tracking individual user interactions with specific sections in LINE OA is challenging due to its backend limitations, as only aggregate user activity data are available. This impedes the establishment of personalized sections, as suggested by the heuristic evaluators in phase 2.

Analysis of phase 3 error records further revealed 2 major sources of confusion (Multimedia Appendix 4). First, users were often unsure whether to seek answers in Health Education Information or Inquiry & Consultation, as both sections were perceived to serve a similar purpose of obtaining CKD-related knowledge. This mismatch between users’ mental models and the platform’s menu structure frequently led them to attempt tasks in the wrong section, causing navigation failures. Second, the design of LINE OA as an instant messaging interface posed inherent challenges: the service menu occupies nearly one-third of the small mobile screen, while new messages push earlier information upward, forcing users to scroll back or reselect menu buttons to revisit prior content. When combined with multilayered menu depth, these constraints increased cognitive load and disorientation, further compounding the likelihood of users getting lost.

To overcome these challenges, several measures have been introduced and others are planned. In response to phase 2 feedback, we incorporated a “return to previous level” option within the Kidney Support Teammate and Inquiry & Consultation sections (Figure 2D), providing a route back when disoriented. Future platform refinements could also consolidate Health Education Information and Inquiry & Consultation into a single section, for instance, by embedding Inquiry & Consultation under Health Education Information to reduce functional overlap and user confusion. Moreover, involving CKD dyads in early stages of prototype development remains essential. Interviews on information needs and common questions could align menu structures more closely with users’ mental models. Finally, harnessing the instant messaging nature of LINE OA, the inclusion of a chatbot could provide more efficient and context-sensitive access to disease-related information through automated dialogue interactions, thereby enhancing overall usability [21].

#### Concerns for Low EARs

Despite our efforts to simplify the design of our digital platform, reducing hierarchies and operational paths, along with manual support and relevant skill teachings, some users, as shown in Table 7, have an activity rate below 50% for logging physiological values (2 users) or writing kidney diaries (4 users). We identified 3 potential reasons for these low EARs, indicating the need for more comprehensive interventions as mentioned in the following sections.

##### Dyads in the Preaction Stages of Change

Observations from Tables5  7 revealed a positive relationship between dyads’ self-reported readiness for lifestyle modification (ie, stages of change) and their execution of daily tasks. The 5 most active dyads, with EARs exceeding 75% for both tasks and having achieved the *Master* kidney health title, were all in the late stages of change (*action* or *maintenance*), while of the other 5 dyads, 3 were in the preaction stages (*contemplation* or *preparation*). Despite daily task reminders, there was no observed increase in activity rates among lower-activity users. Future interventions may focus on leveraging helping relationships with SOs and providing proactive caring phone calls to enhance their motivation for change.

##### Dyads Perceive No Need for Use

Among the previously mentioned 5 dyads with lower activity rates, 2 self-reported being in the late stage of change (*maintenance*). These dyads might have already engaged in regular lifestyle modifications but perceive minimal help from our digital platform and hence feel no necessity in using it. To address this, future implementation could focus on highlighting the platform’s strengths and collaborating with health care providers to promote the platform’s benefits to patients and their caregivers.

##### Dyads Face Usage Obstacles and Perceive High Execution Difficulty

Another possible cause of low EARs is shortcomings in the digital platform’s design and user manual, leading dyads to perceive high cognitive load and execution difficulty. To address this, helping relationships from SOs and providing assistance are crucial. Regular caring and reminder messages, platform design optimization, and personalized teaching during return visits can help address usage obstacles and enhance usability.

Another promising future direction is to assess the feasibility of organizing support groups for CKD dyads, based on our dyadic feedback in phase 1. Support groups may facilitate the process of social liberation, exposing dyads to a supportive social environment for behavior change [42]. Dyads in the preaction stages might find positive support from the group, enhancing their motivation for change. Support groups can also promote community participation through connections and information exchange and allow their efforts to gain recognition and reinforcement from the group. For dyads perceiving minimal help from the digital platform or finding it too challenging to use, they can observe and learn from other dyads in the support groups, enhancing their confidence and skills in usage.

### Lack of Feedback From Health Care Providers

In phase 1 dyad trial use and phase 2 heuristic evaluation, participants highlighted the need for health care provider feedback on user self-monitoring records. However, our digital platform currently lacks feedback mechanisms beyond system-generated physiological trend charts and platform usage reports from the extended app (Figures 3A and 3C). Specifically, feedback from health care providers on dietary, medication, and exercise content in the kidney health diary is absent. As observed in Figure 5, health care providers currently do not interact with CKD dyads through our digital platform and are thus unable to provide feedback on the content of the kidney health diary. This gap may contribute to lower activity rates for the kidney health diary compared with physiological values (Table 7). Workflow integration challenges are common in the development and application of new digital health tools, as they might potentially burden health care providers with additional tasks or even necessitate practice reorganization [15,43].

Recognizing the temporary difficulty in overcoming this challenge, an alternative approach involves compiling CKD dyads’ self-monitoring records into paper-based reports for health care providers. These reports are provided to health care providers on CKD dyads’ follow-up visit days for reference. During consultations, health care providers can then access the extended app with CKD dyads to review the kidney health diary content as needed. While conducting trials to assess the effect of our digital platform on CKD management is imperative, our future work should also tackle workflow integration issues through close collaboration with health care system personnel to promote platform adoption.

### Conclusions

Guided by DDEF and using Agile methods, this study successfully developed, evaluated, and optimized an instant messaging–based digital platform tailored for CKD care across a 3-phase development cycle. The resulting product, the LINE OA “Kidney Lifestyle” and its associated extended app, aims to empower lifestyle modification for CKD dyads. Our investigation uncovered robustly high user acceptance and satisfaction with the platform, evidenced by both self-report ratings and ongoing engagement beyond the study period, lasting over 6 months. The platform’s wide availability, high automation, and cost-effective operational model underscore its potential for scalable application in practical health care settings. While our digital solution shows considerable promise, a feasibility study is the logical next step to evaluate implementation factors and potential impacts on CKD management outcomes. Meanwhile, our future works should also bridge digital divides, enhance usability, foster support groups, and integrate our platform into current health care workflow to maximize its effectiveness and scalability. In sum, this study contributes significantly to the growing field of digital health for CKD management by showcasing the potential of an innovative platform integrated with the LINE instant messaging app.

## Supplementary material

10.2196/73935 Multimedia Appendix 1 User manual.

10.2196/73935 Multimedia Appendix 2 Phase 1 user feedback form.

10.2196/73935 Multimedia Appendix 3 Phase 3 usability testing instrument.

10.2196/73935 Multimedia Appendix 4 Complete dataset and summary tables.

## Abbreviations

API: application programming interface
ASQ: After-Scenario Questionnaire
CKD: chronic kidney disease
CREA: creatinine
DDEF: Digital Dyadic Empowerment Framework
DHI: digital health intervention
EAR: entry activity rate
eGFR: estimated glomerular filtration rate
OA: LINE Official Account
SO: significant other
SUS: System Usability Scale
