# Supplementary material for: A Messaging App Empowering Lifestyle Modification in Chronic Kidney Disease (LINE Official Account “Kidney Lifestyle”): Platform Development and Usability Study
Source: JMIR Hum Factors. 2025 Nov 26;12:e73935. doi: 10.2196/73935 (PMC12661614; doi:10.2196/73935)
Supplement: Multimedia Appendix 1 [file humanfactors-v12-e73935-s001.pdf]

LINE官方帳號
「健腎生活」及擴充App
使用手冊

國家衛生研究院 整合性醫藥衛生科技研究計畫

計畫名稱： 慢性腎臟疾病生活型態調整之數位對偶充能計畫

計畫主持人： 成功大學護理學系 顏妙芬 教授

合作夥伴： AlleyPin 翔評互動

手冊製作人： 研究助理 何俊毅

114年1月7日

打開您的LINE主頁
在**右上方**找到並點選 ⛶ 按鈕

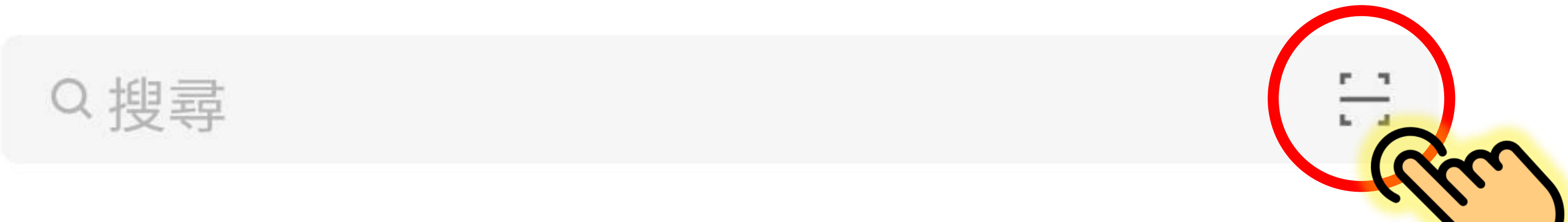

接著掃描下方QR code

歡迎加入LINE官方帳號
「健腎生活」！

使用上遇到任何問題嗎？
歡迎聯絡 06-2353535 轉 5852
找研究助理 何俊毅 / 鄭姿蕊

手冊目錄

一、LINE官方帳號介面說明 5

二、「健腎生活」擴充App介面說明 ... 7

App各項基本功能介紹

① **記錄數值**：登錄我的生理數值 9

② **健腎日記**：撰寫每日健腎日記 11

新增當天日記 13

③ **使用報告**：查看健腎稱號挑戰進度 15

④ **提醒設定**：接收下次回診提醒 17

首次設定提醒 19

給親愛的健腎者：

首先，非常感謝您願意參與本研究計畫！

延緩腎功能下降和洗腎風險，有賴您維持**健康的生活型態**。LINE官方帳號「健腎生活」希望能與您一起努力，**建立居家健康管理習慣**。

如果您對操作3C產品不熟悉甚至有些恐懼，請不用擔心，我們將提供完善教學，這本使用手冊也能幫助您上手。如果您在操作上有任何問題，也歡迎您告訴我們，您的回饋將有助我們改善使用體驗，以造福更多腎友哦！

成功大學護理學系 顏妙芬教授團隊

× AlleyPin翔評互動

關心您

一、LINE官方帳號介面說明

就像您LINE裡面的親朋好友一樣，您可以點選右方畫面中紅色框框的各個按鈕，與LINE官方帳號「健腎生活」進行互動！

1 開啟／關閉「服務選單」(即2)

2 服務選單：共8個按鈕可分別點選

3 傳給您的訊息下方若有按鈕，可點選

4 向左滑動，瀏覽多頁式訊息內容

5 召喚打字鍵盤，可打字並傳送訊息

6 關閉聊天室，返回到LINE的聊天列表

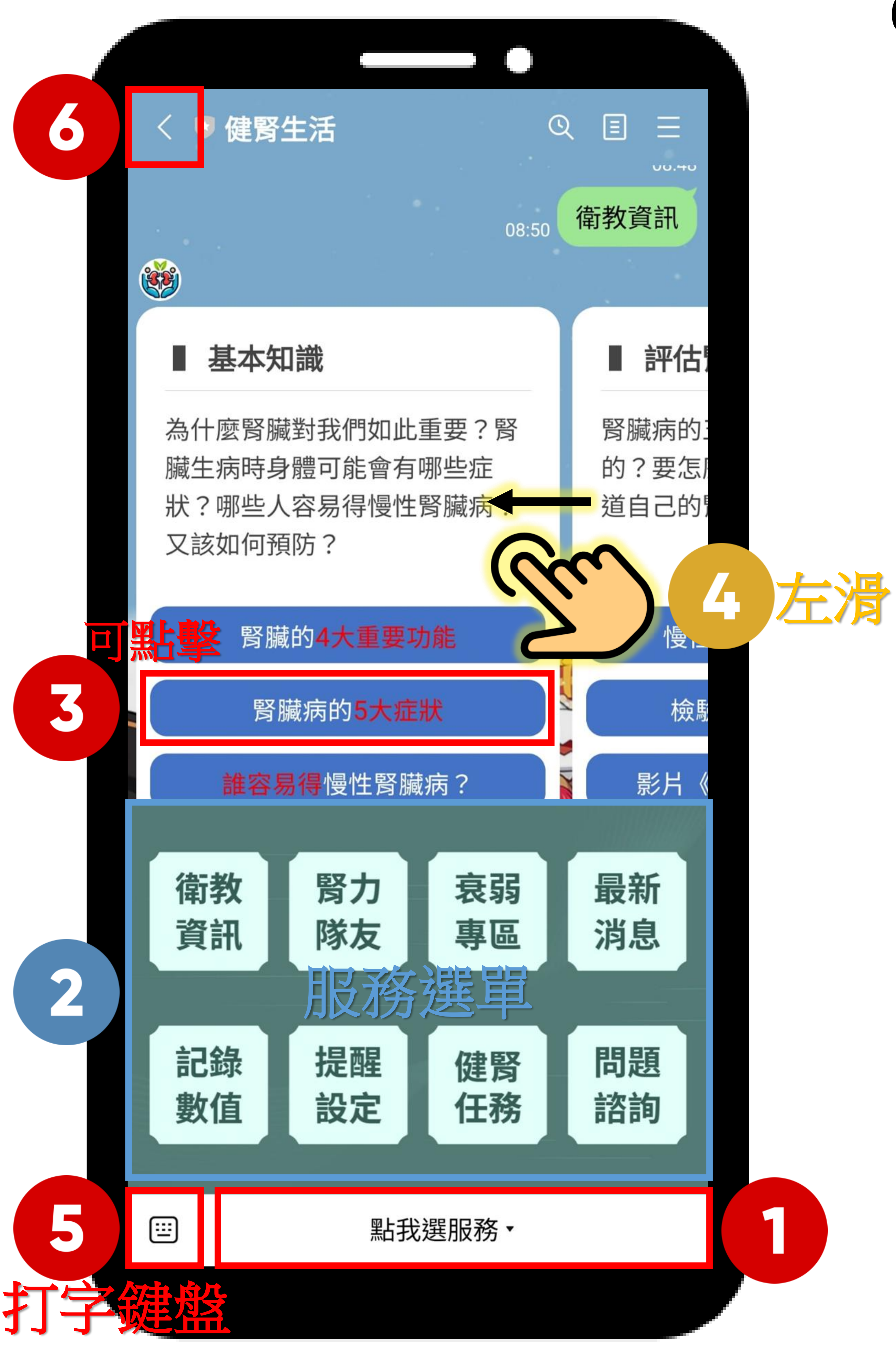
6
健腎生活
衛教資訊
08:50
基本知識
為什麼腎臟對我們如此重要？腎臟生病時身體可能會有哪些症狀？哪些人容易得慢性腎臟病又該如何預防？
評估
可點擊
腎臟的4大重要功能
3
腎臟病的5大症狀
誰容易得慢性腎臟病？
檢
影片
4
左滑
2
衛教資訊
腎力隊友
衰弱專區
最新消息
服務選單
記錄數值
提醒設定
健腎任務
問題諮詢
5
點我選服務
1
打字鍵盤

二、擴充App介面說明

以檢視「血壓記錄」為例，當您登錄過生理數值後，您可以透過點選右方畫面中紅色框框的按鈕，嘗試App的不同功能！

1. 功能選單：共5個按鈕可分別點選
2. 點擊後可進入查看詳細登錄內容
   向上滑動可往下查看較早以前的登錄
3. 點擊右下角可編輯或刪除該筆登錄
4. 重新整理：將資料更新至最新狀態
   當**畫面消失或資料未顯示**可點選這個

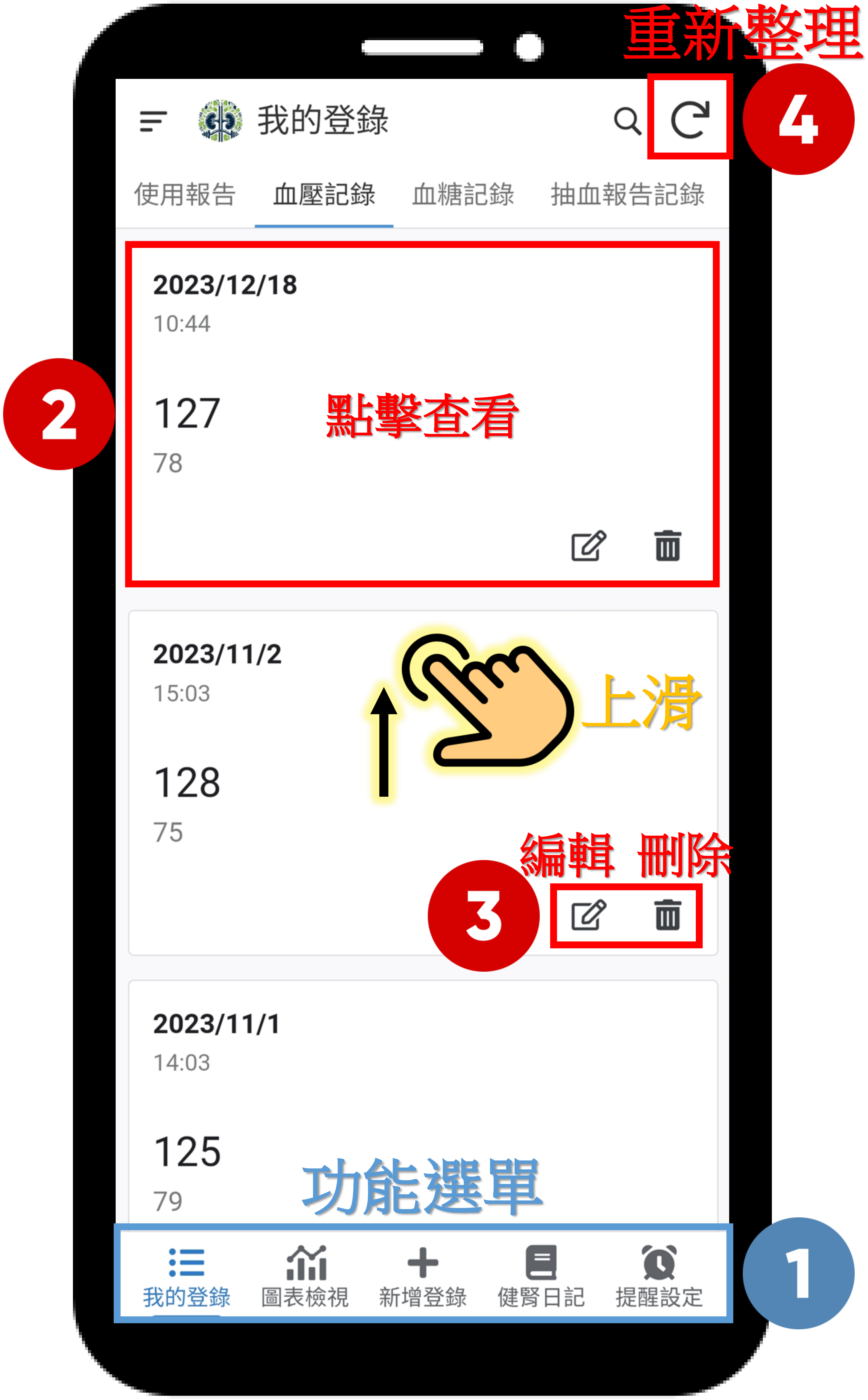
重新整理
4
我的登錄
使用報告
血壓記錄
血糖記錄
抽血報告記錄
2023/12/18
10:44
127
78
點擊查看
2
2023/11/2
15:03
128
75
上滑
編輯 刪除
3
2023/11/1
14:03
125
79
功能選單
我的登錄
圖表檢視
新增登錄
健腎日記
提醒設定
1

① 記錄數值

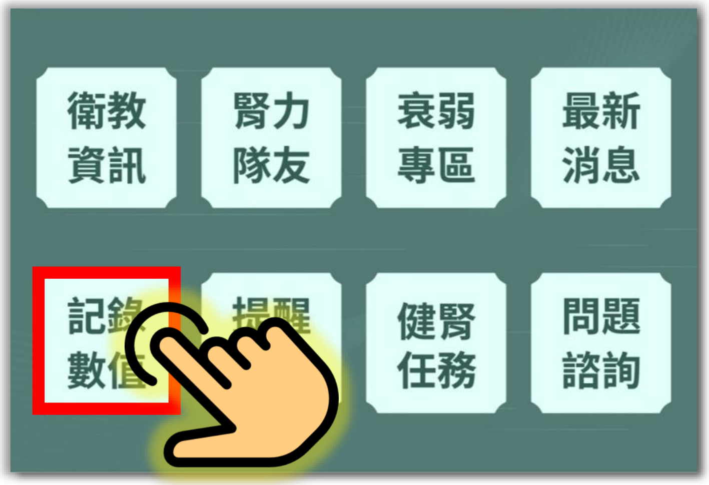

如何開啟如右方 ➔ 的登錄頁面？

- ✓ 方法一：點選LINE服務選單左下角「記錄數值」按鈕
- ✓ 方法二：點選App功能選單中間的「新增登錄」按鈕

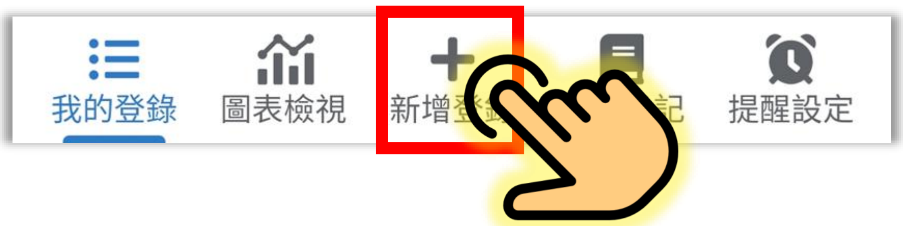

1. 點選格子，召喚數字鍵盤
依序逐格輸入數值 (* 為必填項目)
2. 可直接點選選項 (選取後為藍色)
3. 輸入完畢後，按下儲存。您可於App功能選單點選「圖表檢視」查看報表
4. 猜猜看：這個箭頭代表什麼操作？
_____ (提示：本手冊第6頁的LINE介面)

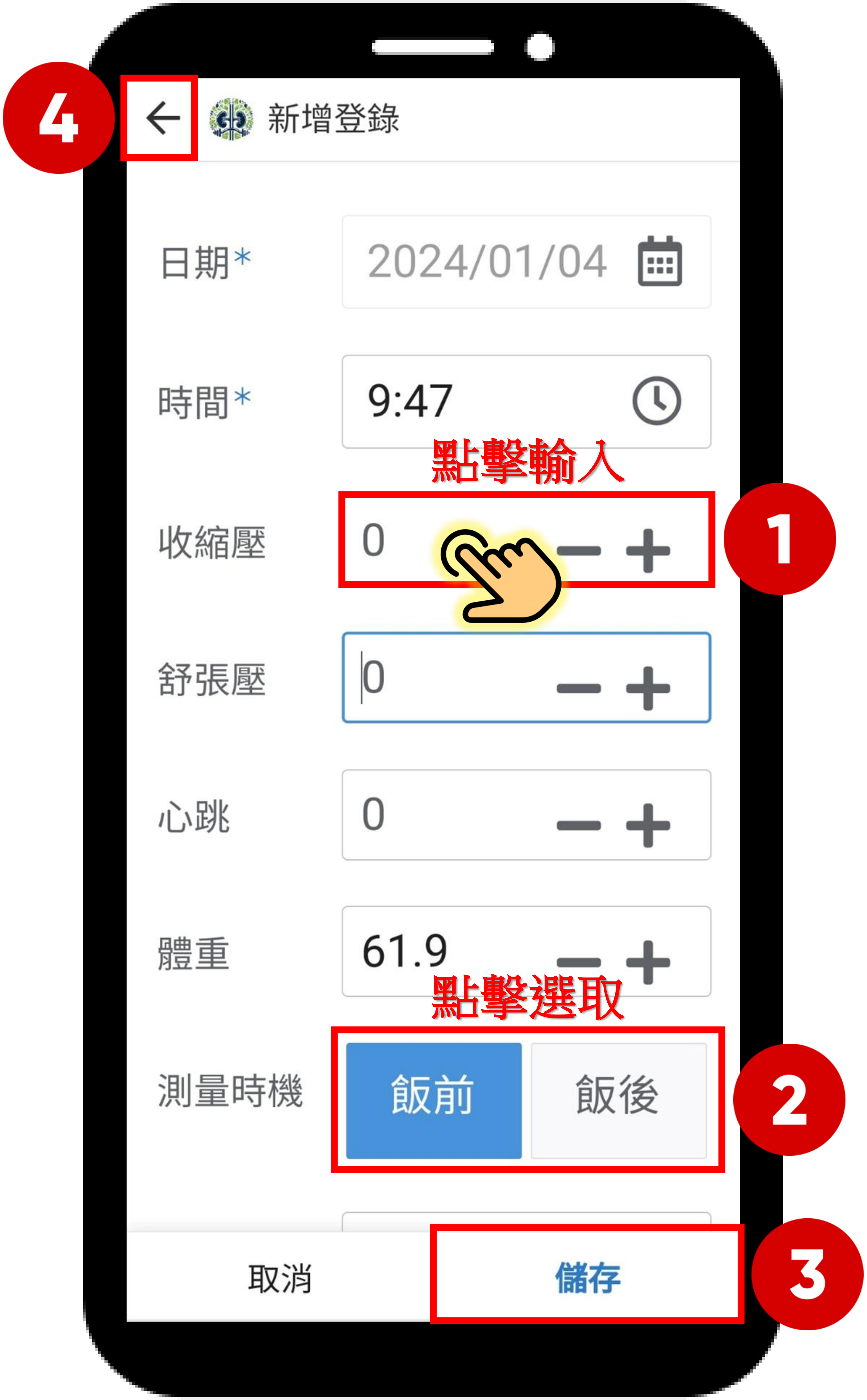
4
新增登錄
日期*
2024/01/04
時間*
9:47
點擊輸入
收縮壓
0
1
舒張壓
0
心跳
0
體重
61.9
點擊選取
測量時機
飯前
飯後
2
取消
儲存
3

於App功能選單點擊

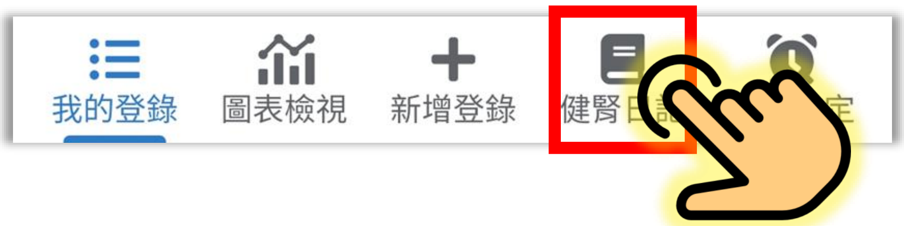

② 健腎日記

每天撰寫一篇健腎日記，記錄三餐飲食、用藥和身體活動情形，方便您掌握自己的生活型態調整狀況，也讓醫護人員能在您回診時根據日記內容給您專業建議！

1. 點選「健腎日記」開啟如右方→畫面
向上滑動可往下查看較早以前的日記

2. 點擊後可進入查看詳細日記內容

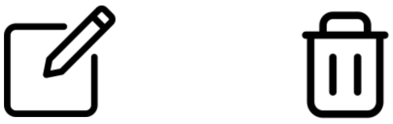

3. 點擊「⋮」可編輯或刪除該篇日記

4. 新增當天日記，開啟如第14頁畫面；
每天只需新增一篇日記，故當天若已新增日記，可透過 3 來修改內容

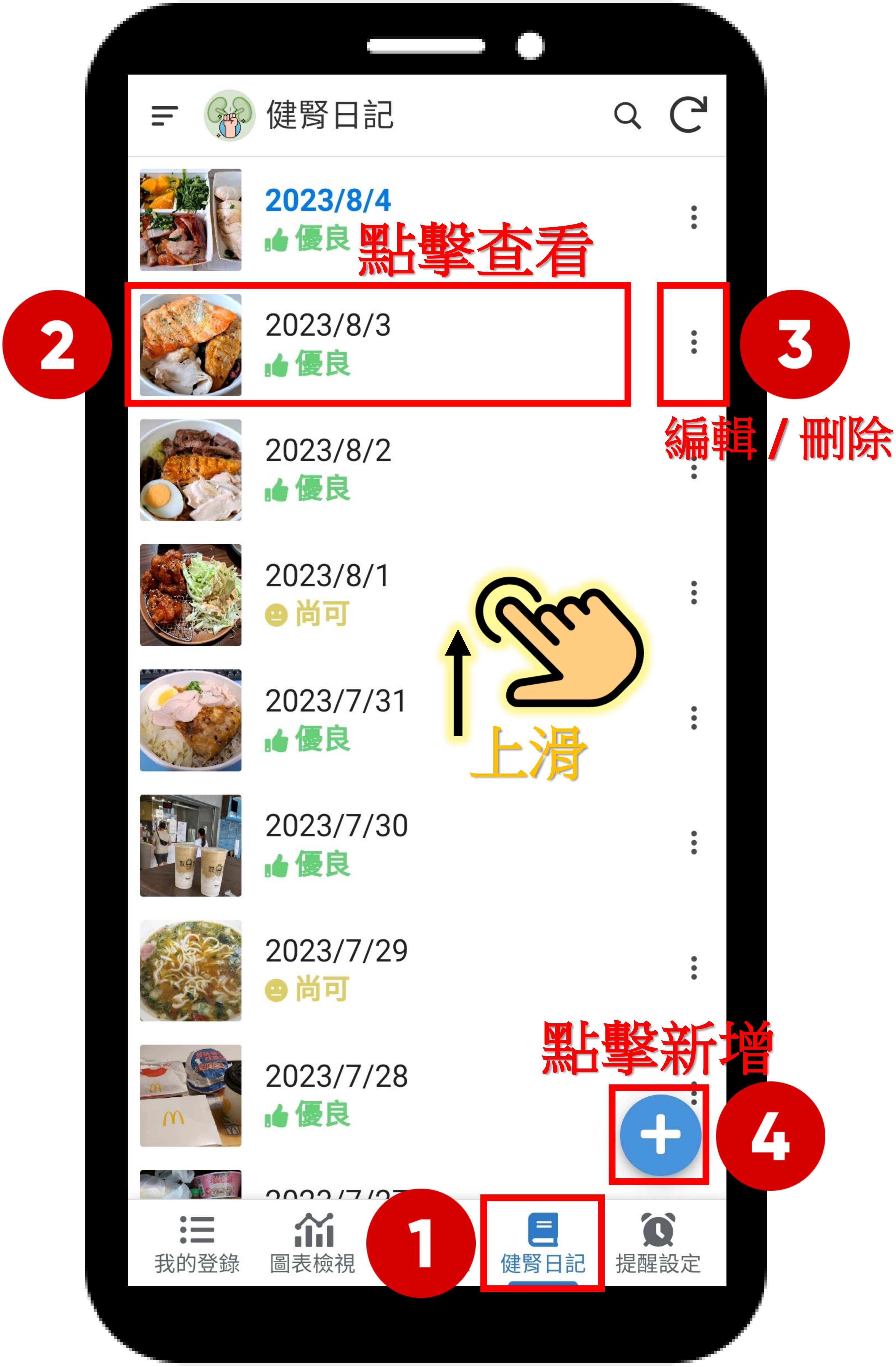

健腎日記
2023/8/4
優良
點擊查看
2
2023/8/3
優良
3
編輯 / 刪除
2023/8/2
優良
2023/8/1
尚可
上滑
2023/7/31
優良
2023/7/30
優良
2023/7/29
尚可
點擊新增
2023/7/28
優良
4
1
我的登錄
圖表檢視
健腎日記
提醒設定

新增當天日記

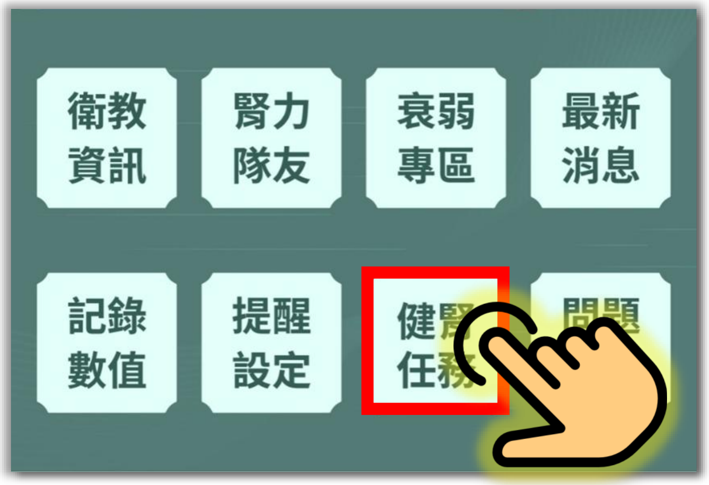

日記內容包含以下三項：

- **三餐飲食**拍照或文字記錄
- 檢核當天的**服藥情形**
- 檢核當天**是否運動**和走路步數

1. 上傳照片：可選擇「**拍攝照片**」直接開啟手機鏡頭拍照餐點；或者「**從相簿選取**」上傳手機中現有的照片
2. 您也可以點擊備註框框，打字輸入該餐的備註 (例如吃了什麼、份量多少)
3. 向上滑動以往下依序記錄 (1) 三餐、(2) 服藥情形 和 (3) 身體活動情形
4. 儲存已撰寫的日記內容

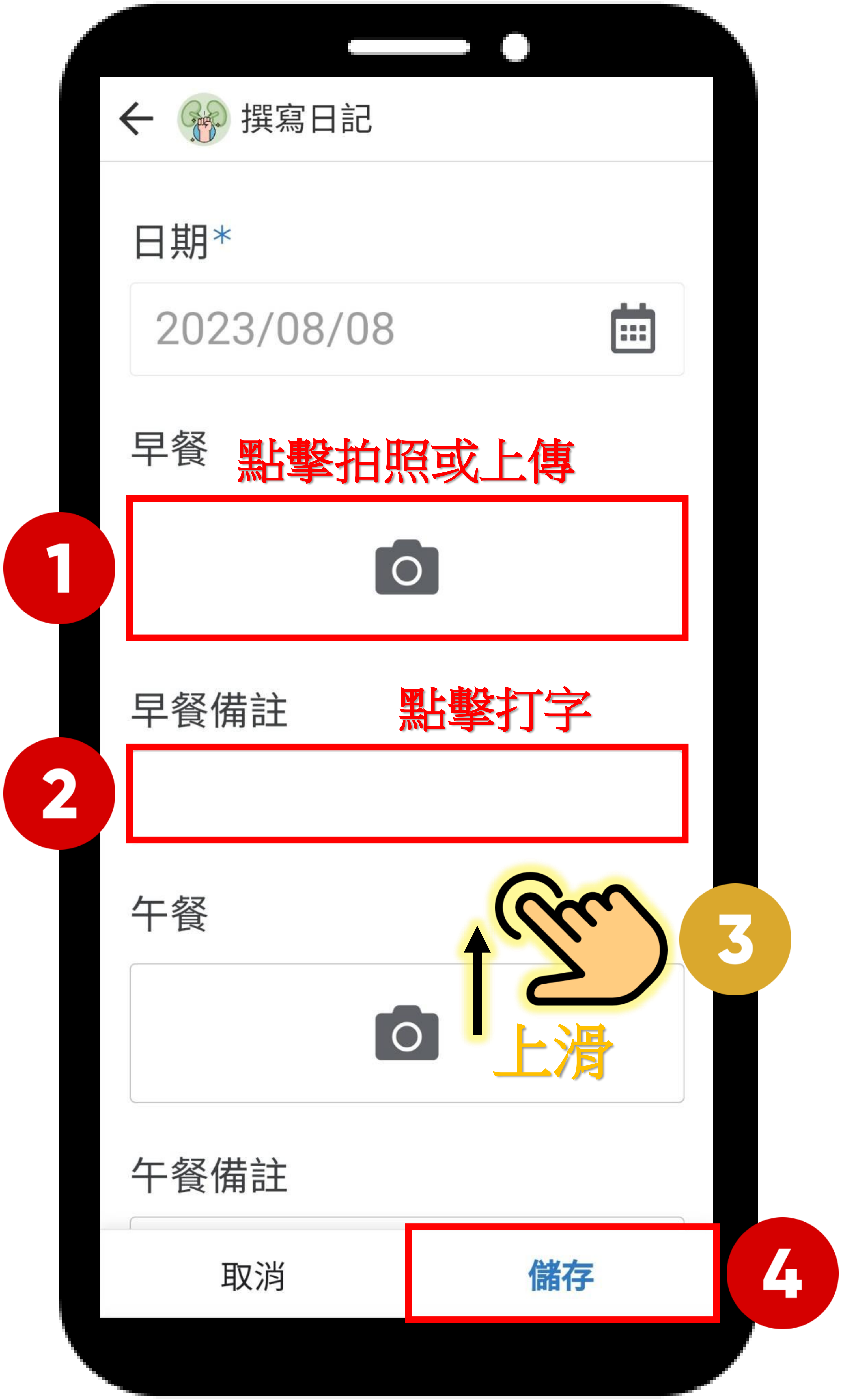
撰寫日記
日期*
2023/08/08
早餐
點擊拍照或上傳
1
早餐備註
點擊打字
2
午餐
3
上滑
午餐備註
取消
儲存
4

於App功能選單點擊

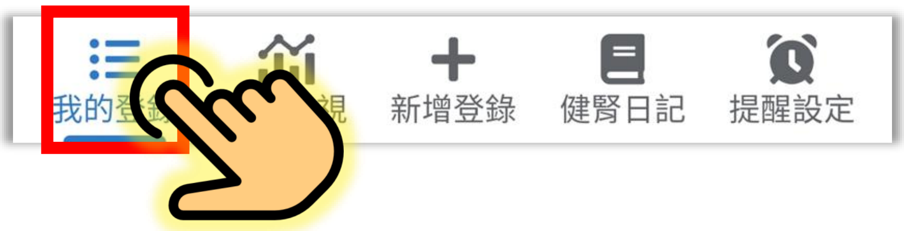

③ 使用報告

1 點選功能選單中最左邊的「我的登錄」查看報告 (如右方 ➔ 畫面)

2 根據您「連續」登錄生理數值和撰寫健腎日記的天數，賦予的健腎稱號

連續3天	健腎新手
連續7天	健腎進階者
連續14天	???
連續30天	健腎大師
連續60天	???

3 您正在挑戰的稱號以及進度百分比

4 紅色鈴鐺提醒您當天尚未完成該項目

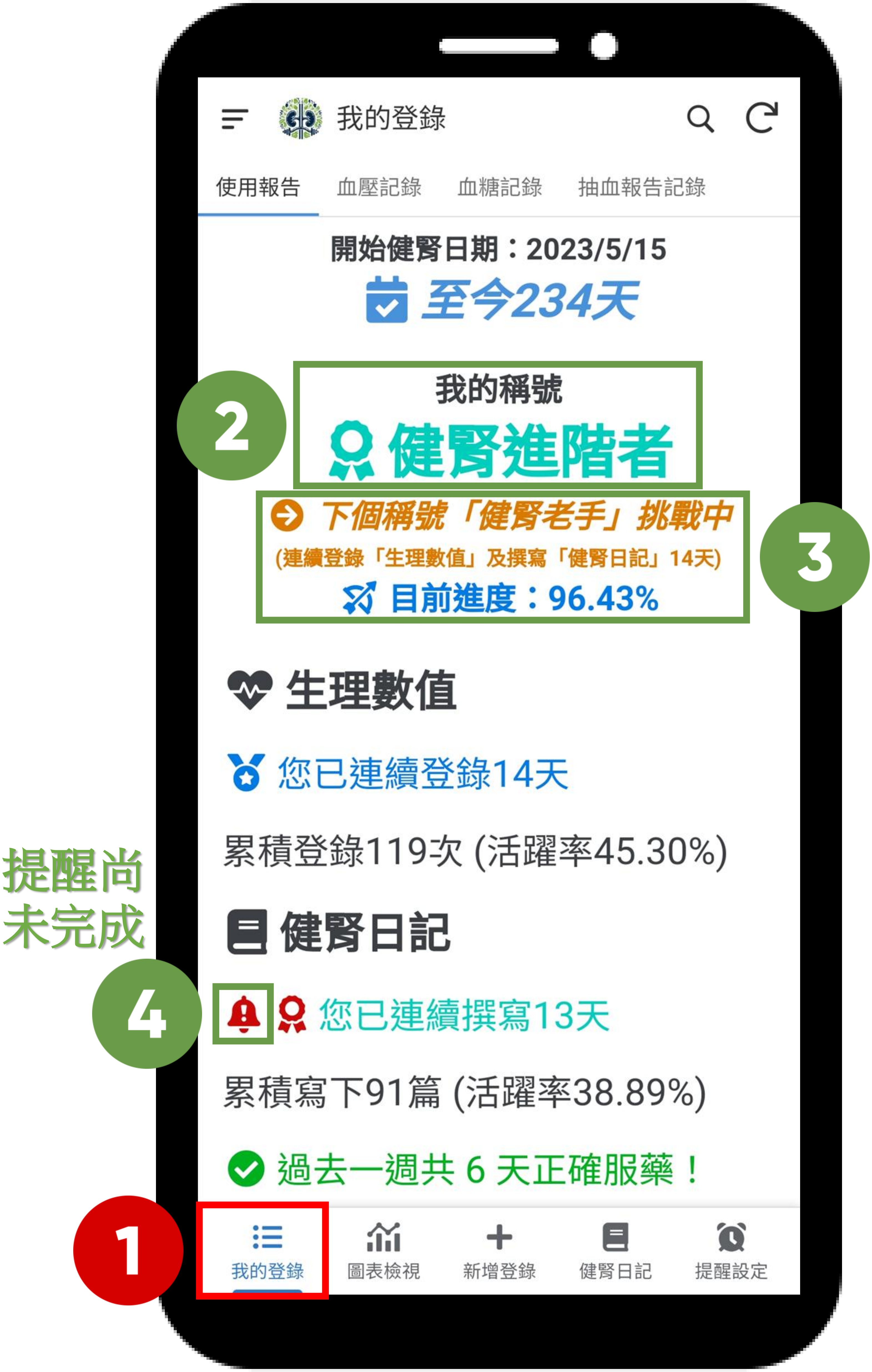

我的登錄
使用報告
血壓記錄
血糖記錄
抽血報告記錄
開始健腎日期：2023/5/15
至今234天
2
我的稱號
健腎進階者
下個稱號「健腎老手」挑戰中
(連續登錄「生理數值」及撰寫「健腎日記」14天)
目前進度：96.43%
3
生理數值
您已連續登錄14天
累積登錄119次 (活躍率45.30%)
健腎日記
提醒尚未完成
4
您已連續撰寫13天
累積寫下91篇 (活躍率38.89%)
過去一週共 6 天正確服藥！
1
我的登錄
圖表檢視
新增登錄
健腎日記
提醒設定

於App功能選單點擊

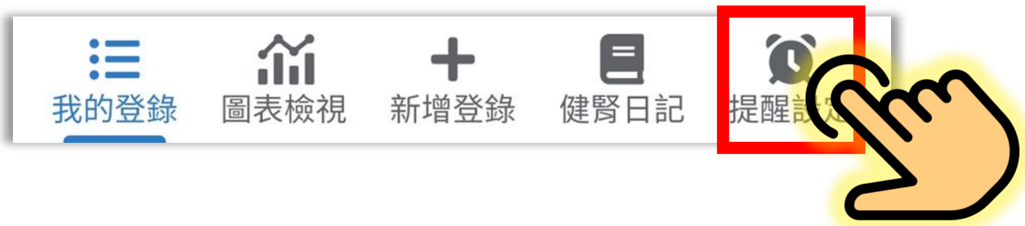

④ 提醒設定

1 點選功能選單中最右邊的「提醒設定」開啟如右方 ➔ 畫面

2 用戶識別碼為您的「身分證字號末四碼＋生日日期四碼」，當您更換裝置時，此號碼將協助我們找回您的資料

3 點選此框框召喚日期時間設定畫面：

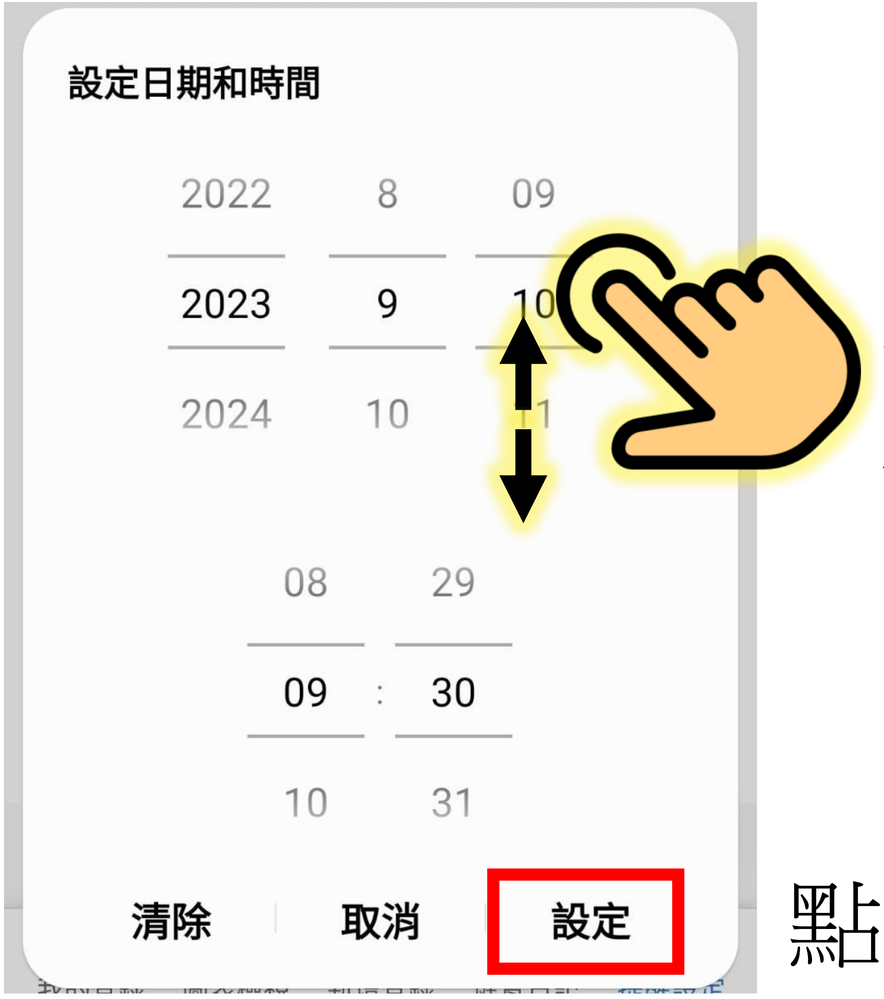

上下滑動以設定日期時間

點選「設定」儲存

4 若您點選 1 未出現如右方 ➔ 畫面，請點選這裡並按「首次設定提醒」

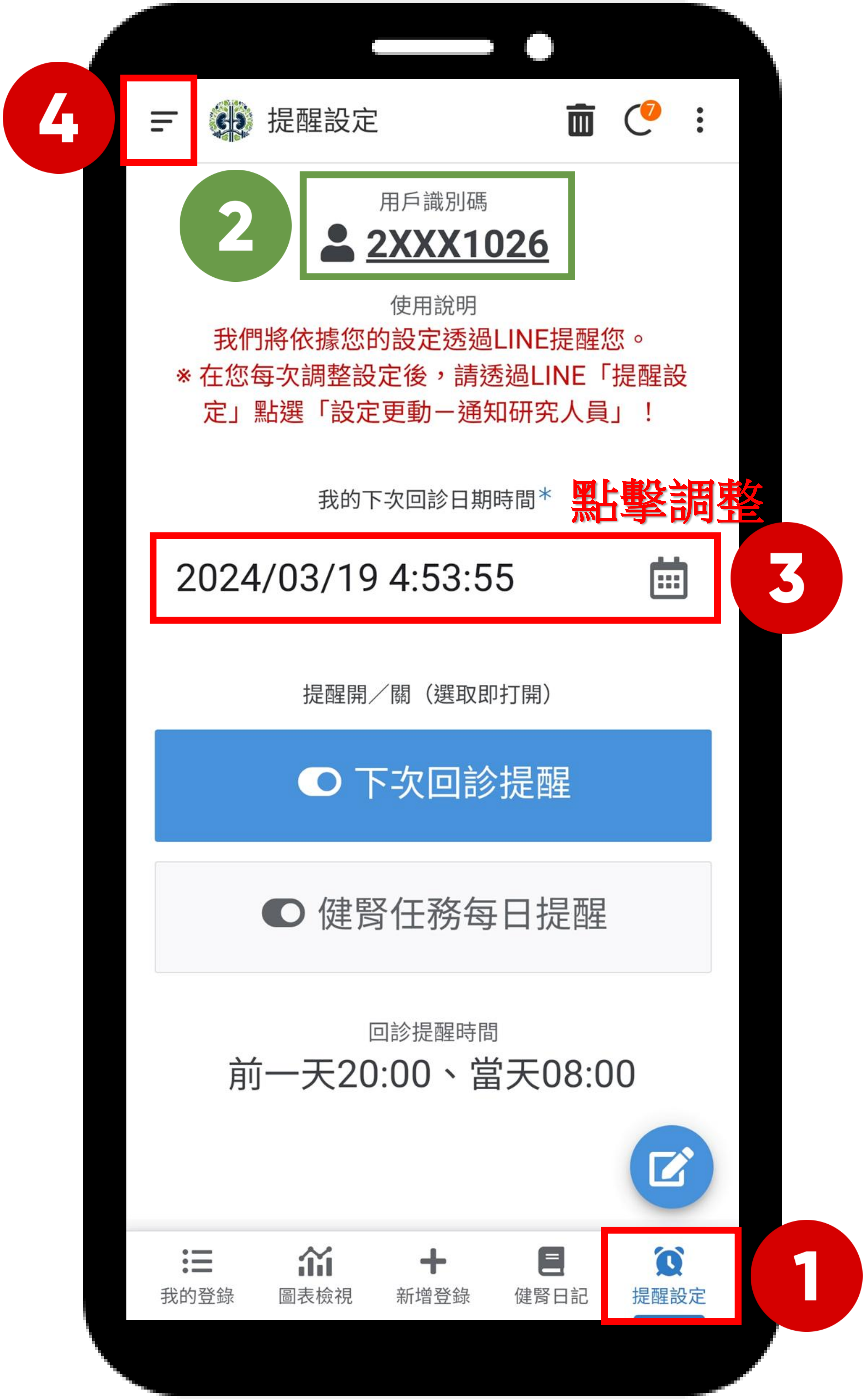
4
提醒設定
2
用戶識別碼
2XXX1026
使用說明
我們將依據您的設定透過LINE提醒您。
※ 在您每次調整設定後，請透過LINE「提醒設定」點選「設定更動－通知研究人員」！
我的下次回診日期時間*
點擊調整
2024/03/19 4:53:55
3
提醒開／關（選取即打開）
下次回診提醒
健腎任務每日提醒
回診提醒時間
前一天20:00、當天08:00
我的登錄
圖表檢視
新增登錄
健腎日記
提醒設定
1

首次設定提醒

在您按下「 ☰ 」以後，請進一步點選「**首次設定提醒**」開啟如右方 ➔ 畫面

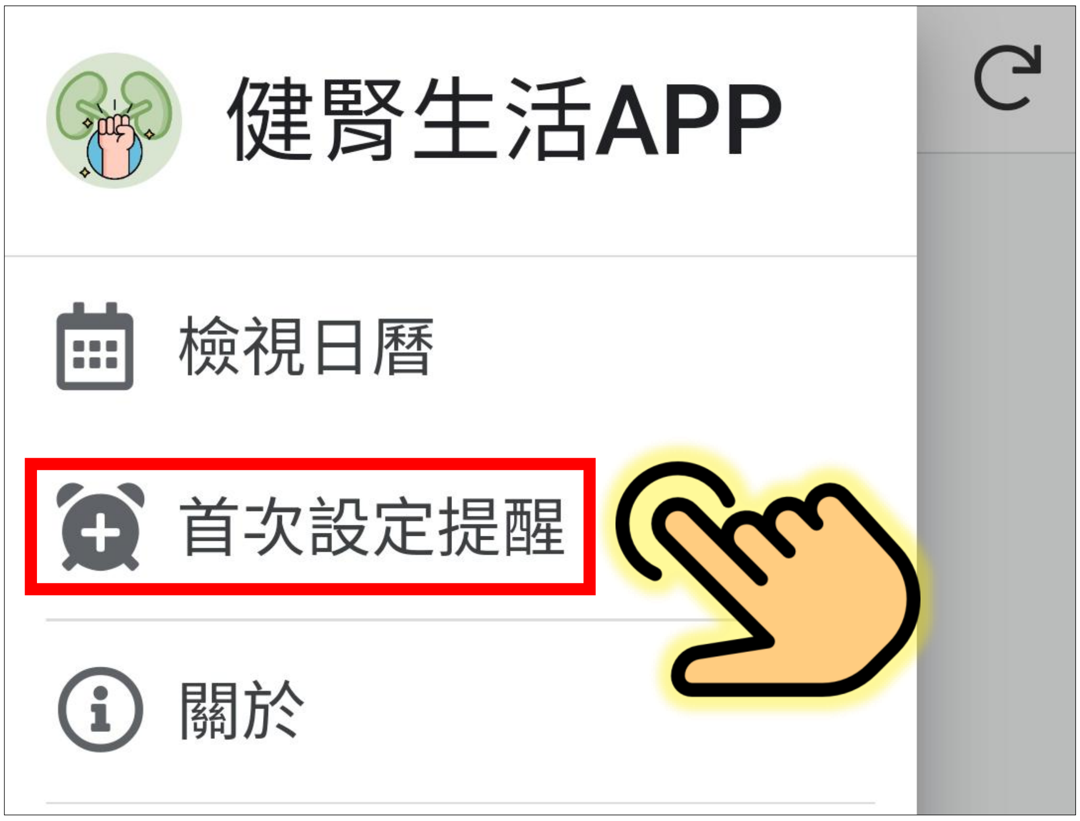

1. 接著，輸入用戶識別碼（您的「身分證字號末四碼＋生日日期四碼」）
   用戶識別碼範例：45XX0629（共8碼）
2. 點選框框，設定下次回診日期時間
3. 輸入完畢後，按下儲存。此時再點選「**提醒設定**」即可開啟如第18頁畫面

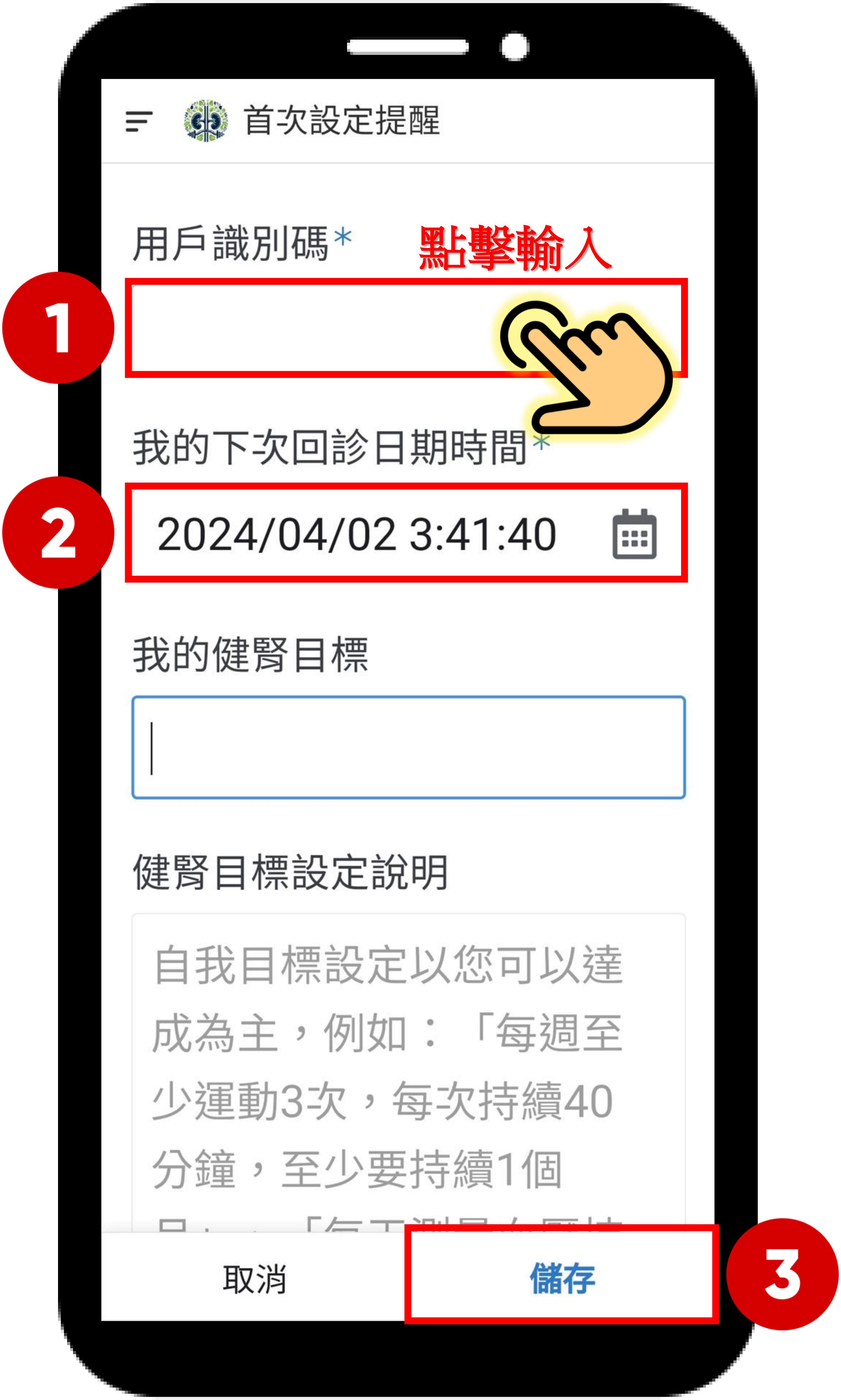
首次設定提醒
用戶識別碼*
點擊輸入
1
我的下次回診日期時間*
2
2024/04/02 3:41:40
我的健腎目標
健腎目標設定說明
自我目標設定以您可以達
成為主，例如：「每週至
少運動3次，每次持續40
分鐘，至少要持續1個
取消
儲存
3
