# Supplementary material for: A Messaging App Empowering Lifestyle Modification in Chronic Kidney Disease (LINE Official Account “Kidney Lifestyle”): Platform Development and Usability Study
Source: JMIR Hum Factors. 2025 Nov 26;12:e73935. doi: 10.2196/73935 (PMC12661614; doi:10.2196/73935)
Supplement: Multimedia Appendix 2 [file humanfactors-v12-e73935-s002.pdf]

數位對偶充能計畫第二年「健腎生活」焦點團體討論使用者回饋表

病人姓名：____________ 重要關鍵人姓名：____________ 編號：____________

一、各項功能評估

1. 衛教資訊（LINE）

功能說明：多頁式訊息提供基本腎臟（病）知識、評估腎功能、腎友飲食及運動指南

<table>
<tr><th colspan="2">請試著回答下列問題：</th><th>您的回答</th></tr>
<tr><td rowspan="2">1-1</td><td colspan="2">此功能在畫面上，有什麼不清楚的地方嗎？ （有／沒有）</td></tr>
<tr><td>若有不清楚，具體是指哪些地方呢？</td><td>例如：字太小、顏色太花不容易掌握重點</td></tr>
<tr><td rowspan="2">1-2</td><td colspan="2">此功能在操作上是否簡單易用？ （是／否）</td></tr>
<tr><td>若您感到操作困難，您覺得原因是？</td><td>例如：按鈕太密集容易點錯、點進去會迷路</td></tr>
<tr><td rowspan="3">1-3</td><td colspan="2">您是否喜歡使用此功能？ （是／否）</td></tr>
<tr><td>若您喜歡，歡迎告訴我們原因！</td><td></td></tr>
<tr><td>若您不喜歡，我們該如何改善它？</td><td></td></tr>
<tr><td rowspan="2">1-4</td><td colspan="2">您未來會持續使用這個功能嗎？ （會／不會）</td></tr>
<tr><td>無論會不會，歡迎告訴我們原因！</td><td></td></tr>
<tr><td>1-5</td><td>您對「衛教資訊」的滿意度評分為？</td><td>最低 1 顆星、最高 5 顆星
☆ ☆ ☆ ☆ ☆</td></tr>
</table>

2. 腎力隊友（LINE）

功能說明：多頁式訊息提供照顧者（重要關鍵人）支持資源

請試著回答下列問題：		您的回答
2-1	此功能在畫面上，有什麼不清楚的地方嗎？　（有／沒有）	
	若有不清楚，具體是指哪些地方呢？	例如：字太小、顏色太花不容易掌握重點
2-2	此功能在操作上是否簡單易用？　（是／否）	
	若您感到操作困難，您覺得原因是？	例如：按鈕太密集容易點錯、點進去會迷路
2-3	您是否喜歡使用此功能？　（是／否）	
	若您喜歡，歡迎告訴我們原因！	
	若您不喜歡，我們該如何改善它？	
2-4	您未來會持續使用這個功能嗎？　（會／不會）	
	無論會不會，歡迎告訴我們原因！	
2-5	您對「腎力隊友」的滿意度評分為？	最低 1 顆星、最高 5 顆星<br>☆ ☆ ☆ ☆ ☆

3. 記錄數值（LINE+擴充 App）（搭配手冊第 9-10 頁）

功能說明：使用者登錄血壓、心跳、體重與血糖，系統記錄並生成報表回傳

請試著回答下列問題：		您的回答
3-1	此功能在畫面上，有什麼不清楚的地方嗎？　（有／沒有）	
	若有不清楚，具體是指哪些地方呢？	例如：字太小
3-2	此功能在操作上是否簡單易用？　（是／否）	
	若您感到操作困難，您覺得原因是？	例如：框框太小、點進去會迷路
3-3	您是否喜歡使用此功能？　（是／否）	
	若您喜歡，歡迎告訴我們原因！	
	若您不喜歡，我們該如何改善它？	
3-4	您未來會持續使用這個功能嗎？　（會／不會）	
	無論會不會，歡迎告訴我們原因！	
3-5	您對「記錄數值」的滿意度評分為？	最低 1 顆星、最高 5 顆星<br>☆ ☆ ☆ ☆ ☆

4. 提醒設定（LINE+擴充 App）（搭配手冊第 17-20 頁）

功能說明：使用者登錄下次回診日期時間，系統將於前 1 天與 1 小時前傳訊息提醒

請試著回答下列問題：		您的回答
4-1	此功能在畫面上，有什麼不清楚的地方嗎？ （有／沒有）	
	若有不清楚，具體是指哪些地方呢？	例如：字太小
4-2	此功能在操作上是否簡單易用？ （是／否）	
	若您感到操作困難，您覺得原因是？	例如：框框太小、無法設定正確日期時間
4-3	您是否喜歡使用此功能？ （是／否）	
	若您喜歡，歡迎告訴我們原因！	
	若您不喜歡，我們該如何改善它？	
4-4	您未來會持續使用這個功能嗎？ （會／不會）	
	無論會不會，歡迎告訴我們原因！	
4-5	您對「提醒設定」的滿意度評分為？	最低 1 顆星、最高 5 顆星<br>☆ ☆ ☆ ☆ ☆

5. 健腎任務（LINE+擴充 App）（搭配手冊第 11-16 頁）

功能說明：每日完成登錄「記錄數值」與撰寫「健腎日記」，並挑戰健腎稱號

請試著回答下列問題：		您的回答
5-1	此功能在畫面上，有什麼不清楚的地方嗎？　（有／沒有）	
	若有不清楚，具體是指哪些地方呢？	例如：字太小
5-2	此功能在操作上是否簡單易用？　（是／否）	
	若您感到操作困難，您覺得原因是？	例如：上傳照片有困難
5-3	您是否喜歡使用此功能？　（是／否）	
	若您喜歡，歡迎告訴我們原因！	
	若您不喜歡，我們該如何改善它？	
5-4	您未來會持續使用這個功能嗎？　（會／不會）	
	無論會不會，歡迎告訴我們原因！	
5-5	您對「健腎任務」的滿意度評分為？	最低 1 顆星、最高 5 顆星<br>☆ ☆ ☆ ☆ ☆

6. 問題諮詢（LINE）

功能說明：多頁式訊息提供慢性腎臟病常見問答，若仍有問題提供諮詢管道

請試著回答下列問題：		您的回答
6-1	此功能在畫面上，有什麼不清楚的地方嗎？　（有／沒有）	
	若有不清楚，具體是指哪些地方呢？	例如：字太小、顏色太花不容易掌握重點
6-2	此功能在操作上是否簡單易用？　（是／否）	
	若您感到操作困難，您覺得原因是？	例如：按鈕太密集容易點錯、點進去會迷路
6-3	您是否喜歡使用此功能？　（是／否）	
	若您喜歡，歡迎告訴我們原因！	
	若您不喜歡，我們該如何改善它？	
6-4	您未來會持續使用這個功能嗎？　（會／不會）	
	無論會不會，歡迎告訴我們原因！	
6-5	您對「問題諮詢」的滿意度評分為？	最低 1 顆星、最高 5 顆星<br>☆ ☆ ☆ ☆ ☆

二、整體功能評估

請試著回答下列問題：		您的回答
7-1	您是否曾使用過其他任何管理慢性腎臟病的應用程式／網站？（有／沒有）	
	您喜歡或不喜歡它們的哪些方面？	
7-2	**請針對「健腎生活」LINE 官方帳號與擴充 App 給予整體評價**	
	您對「健腎生活」的整體滿意度？	最低 1 顆星、最高 5 顆星<br>☆ ☆ ☆ ☆ ☆
	您願意持續使用「健腎生活」幫助您管理慢性腎臟病嗎？（願意／不願意）	
