# Supplementary material for: A Messaging App Empowering Lifestyle Modification in Chronic Kidney Disease (LINE Official Account “Kidney Lifestyle”): Platform Development and Usability Study
Source: JMIR Hum Factors. 2025 Nov 26;12:e73935. doi: 10.2196/73935 (PMC12661614; doi:10.2196/73935)
Supplement: Multimedia Appendix 3 [file humanfactors-v12-e73935-s003.pdf]

DDEP 計畫第二年 Step 3 收案記錄表

對偶姓名：＿＿＿＿＿＿＿、＿＿＿＿＿＿＿＿　編號：＿＿＿＿＿

任務 1 情境

以下是您今天回診時量血壓和抽血報告的數值：

收縮壓	125
舒張壓	78
心跳	65
肌酸酐（CREA)	1.4　mg/dL

請您嘗試將以上數值登錄於我們的數位平台。

任務結束後，請回答以下問題

整體而言，您對於完成這項任務的：	非常不同意 1	2	3	4	5	6	非常同意 7
「容易程度」感到滿意	○	○	○	○	○	○	○
「花費時間」感到滿意	○	○	○	○	○	○	○
「使用手冊提供的幫助」感到滿意	○	○	○	○	○	○	○

任務 2 情境

您想了解自己目前在慢性腎臟病第幾期。但您發現這次的抽血報告中未提供您的「腎絲球過濾率 (eGFR)」數值，要用「肌酸酐 (CREA)」(1.4 mg/dL) 結合您的「性別」和「年齡」來換算。

請您嘗試探索我們的數位平台，算出自己的 eGFR 是多少。

答：我的 eGFR 大約是__________，屬於慢性腎臟病第______期。

任務結束後，請回答以下問題

整體而言，您對於完成這項任務的：	非常不同意<br>1	2	3	4	5	6	非常同意<br>7
「容易程度」感到滿意	◯	◯	◯	◯	◯	◯	◯
「花費時間」感到滿意	◯	◯	◯	◯	◯	◯	◯
「使用手冊提供的幫助」感到滿意	◯	◯	◯	◯	◯	◯	◯

任務 3 情境

以下是您下次回診的日期和時間：

2023 年 12 月 28 日下午診 建議報到時間 14:10

請將以上資訊更新於我們的數位平台，並透過平台通知研究人員。

任務結束後，請回答以下問題

整體而言，您對於完成這項任務的：	非常不同意<br>1	2	3	4	5	6	非常同意<br>7
「容易程度」感到滿意	◯	◯	◯	◯	◯	◯	◯
「花費時間」感到滿意	◯	◯	◯	◯	◯	◯	◯
「使用手冊提供的幫助」感到滿意	◯	◯	◯	◯	◯	◯	◯

任務 4 情境

您想了解「低血糖」會出現哪些症狀。

請嘗試探索我們的數位平台，找到低血糖的 7 種可能症狀。

答：當血糖值小於 __________ mg/dL 時，可能出現

頭暈、_____________、發抖、無力、飢餓、___________、昏倒

任務結束後，請回答以下問題

整體而言，您對於完成這項任務的：	非常不同意<br>1	2	3	4	5	6	非常同意<br>7
「容易程度」感到滿意	○	○	○	○	○	○	○
「花費時間」感到滿意	○	○	○	○	○	○	○
「使用手冊提供的幫助」感到滿意	○	○	○	○	○	○	○

任務 5 情境

以下是您最近一餐的飲食內容：

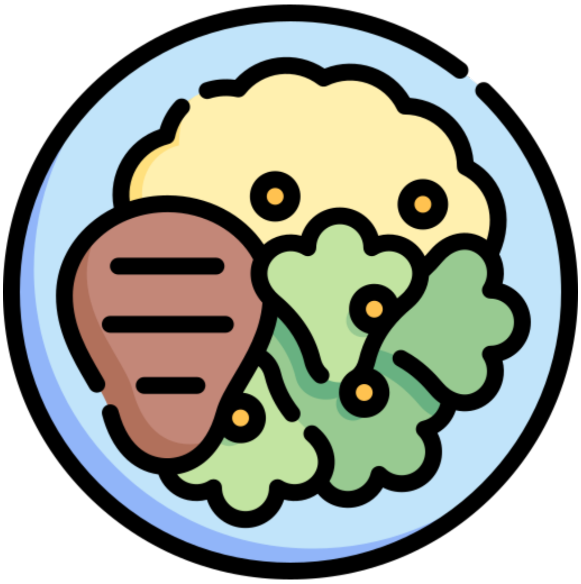

白飯半碗、青江菜 2 份、雞腿肉 1 份

請透過拍照或文字備註方式，將以上飲食記錄於我們的數位平台。

備註：若您以拍照方式記錄，請直接拍攝上方圖片。

任務結束後，請回答以下問題

整體而言，您對於完成這項任務的：	非常不同意 1	2	3	4	5	6	非常同意 7
「容易程度」感到滿意	○	○	○	○	○	○	○
「花費時間」感到滿意	○	○	○	○	○	○	○
「使用手冊提供的幫助」感到滿意	○	○	○	○	○	○	○

任務 6 情境

您的家人 (伴侶或子女) 最近正在努力調整自己的生活型態。

您希望知道一些支持他改變的方法。

請您嘗試探索我們的數位平台，寫出以下具體行為對應的支持策略。

具體行為 1

稱讚對方最近很努力在注意飲食。

答：______________________________ (6 個字)

具體行為 2

和對方一起到附近超市採購不騎車、開車，改用走路的。

答：______________________________ (6 個字)

任務結束後，請回答以下問題

整體而言，您對於完成這項任務的：	非常不同意<br>1	2	3	4	5	6	非常同意<br>7
「容易程度」感到滿意	○	○	○	○	○	○	○
「花費時間」感到滿意	○	○	○	○	○	○	○
「使用手冊提供的幫助」感到滿意	○	○	○	○	○	○	○

整體回饋，請試著回答下列問題

對偶姓名：__________________、__________________ 編號：_____________________

請問您對於 LINE 官方帳號「健腎生活」與擴充 App 的想法是：

系統可用性量表	非常不同意 1	2	3	4	非常同意 5
1.我會想經常使用	○	○	○	○	○
2.我覺得操作上太過複雜	○	○	○	○	○
3.我覺得使用起來很簡單	○	○	○	○	○
4.我覺得我需要有人協助我才能使用	○	○	○	○	○
5.我認為各項功能整合得很好	○	○	○	○	○
6.我認為有很多不一致的地方	○	○	○	○	○
7.我覺得大多數人們能快速學會使用	○	○	○	○	○
8.我覺得使用起來很麻煩	○	○	○	○	○
9.我對於使用此平台很有信心	○	○	○	○	○
10.我需要先學習許多東西才能使用此平台	○	○	○	○	○
